# TALENs facilitate targeted genome editing in human cells with high specificity and low cytotoxicity

**DOI:** 10.1093/nar/gku305

**Published:** 2014-05-03

**Authors:** Claudio Mussolino, Jamal Alzubi, Eli J. Fine, Robert Morbitzer, Thomas J. Cradick, Thomas Lahaye, Gang Bao, Toni Cathomen

**Affiliations:** 1 Institute for Cell and Gene Therapy, University Medical Center Freiburg, 79106 Freiburg, Germany; 2 Center for Chronic Immunodeficiency, University Medical Center Freiburg, 79108 Freiburg, Germany; 3 Institute of Experimental Hematology, Hannover Medical School, 30625 Hannover, Germany; 4 Department of Biomedical Engineering, Georgia Institute of Technology and Emory University, Atlanta, GA 30332, USA; 5 Institute of Genetics, Ludwig-Maximilians-University Munich, 82152 Martinsried, Germany; 6 Center for Plant Molecular Biology, Eberhard-Karls-University, 72076 Tübingen, Germany

## Abstract

Designer nucleases have been successfully employed to modify the genomes of various model organisms and human cell types. While the specificity of zinc-finger nucleases (ZFNs) and RNA-guided endonucleases has been assessed to some extent, little data are available for transcription activator-like effector-based nucleases (TALENs). Here, we have engineered TALEN pairs targeting three human loci (*CCR5, AAVS1* and *IL2RG*) and performed a detailed analysis of their activity, toxicity and specificity. The TALENs showed comparable activity to benchmark ZFNs, with allelic gene disruption frequencies of 15–30% in human cells. Notably, TALEN expression was overall marked by a low cytotoxicity and the absence of cell cycle aberrations. Bioinformatics-based analysis of designer nuclease specificity confirmed partly substantial off-target activity of ZFNs targeting *CCR5* and *AAVS1* at six known and five novel sites, respectively. In contrast, only marginal off-target cleavage activity was detected at four out of 49 predicted off-target sites for *CCR5-* and *AAVS1*-specific TALENs. The rational design of a *CCR5*-specific TALEN pair decreased off-target activity at the closely related *CCR2* locus considerably, consistent with fewer genomic rearrangements between the two loci. In conclusion, our results link nuclease-associated toxicity to off-target cleavage activity and corroborate TALENs as a highly specific platform for future clinical translation.

## INTRODUCTION

Dimeric designer nucleases, such as zinc-finger nucleases (ZFNs) and transcription activator-like effector nucleases (TALENs), have become increasingly popular for targeted genome modification in the last decade (1–3). From the pioneering studies of Kim *et al.* in 1996 (4), significant advancements in the design process of ZFNs and TALENs and a better understanding of parameters determining their activity and toxicity (5,6) have propelled the use of these nucleases from reverse genetics studies in model organisms to their application in human gene therapy (7). These protein-based nucleases are composed of specific DNA binding domains that direct the non-specific *Fok*I endonuclease domain to a predetermined target site (8,9). The resulting DNA double strand break (DSB) can be harnessed to introduce permanent genetic modification by activating one of the two major cellular DSB repair pathways, non-homologous end joining (NHEJ) or homology-directed repair (1). While ZFNs represent the best-characterized class of designer nucleases today, their widespread use has somewhat been hampered by their limited targeting range and the time-consuming methodologies needed to generate ZFNs with novel DNA binding properties (2). TALENs, on the other hand, are easier to engineer and seem to combine high cleavage activity with low cytotoxicity, features that have promoted TALENs as a powerful genome engineering tool in the last two years (3).

The modular DNA recognition domain of transcription activator-like effectors (TALEs) was originally found in natural transcription factors encoded by pathogenic bacteria of the genus *Xanthomonas* (10,11) and more recently in *Ralstonia solanacearum* (12). *Xanthomonas* TALEs are the most widely used in the genome engineering field. Each module within their DNA binding domain consists of a conserved stretch of typically 34 residues that mediates the interaction with a single nucleotide via a di-residue in positions 12 and 13, called the ‘repeat variable di-residues’ (RVDs) (10,11). Modules with different specificities can be fused into tailored arrays without the context-dependency issues that represent the major limitation for the generation of zinc-finger arrays. Hence, this simple ‘one module to one nucleotide’ cypher makes the generation of TALENs with novel specificities rapid and affordable (13,14).

A compelling alternative to ZFNs and TALENs are RNA-guided endonucleases (RGNs) that have quickly developed into an easy and versatile tool for genome engineering (15). They are based on natural RGNs used by bacteria and archaea as a defense system against invading exogenous DNA and consist of the Cas9 cleavage enzyme complexed to a guide RNA (gRNA) strand that directs the enzyme to a ∼20 nt long target site (16). Exchanging specific portions of the gRNA molecule allows scientists to re-direct the Cas9 cleavage activity to user-defined sequences (17).

All of the above described designer nuclease platforms have shown great potential for genome surgery in complex organisms and have been employed with remarkable success to modify genes in a variety of species (1,3,15), including human stem cells (18–23). Notably, ZFNs have been successfully applied in clinical trials for the *ex vivo* modification of patient derived CD4^+^ T cells to generate transplantable HIV-resistant cells by specific disruption of the viral co-receptor *CCR5* (7,24,25). On the other hand, genome-wide assessment of the specificity of the ZFNs employed in these studies revealed a non-trivial degree of off-target cleavage (26,27). Similarly, RGNs have shown high frequency of off-target mutagenesis that, at least in its current form, may hamper their use in therapeutic applications (28–32). A few studies have reported that TALENs can be generated with similar activities as ZFNs (33–36). Moreover, TALENs seem to be better tolerated both in human cell lines and rats (36,37); however, whether ‘better tolerability’ correlates with higher specificity and/or lower off-target cleavage activity has not been addressed in detail yet. High-throughput methods that have been used to profile off-target activities of ZFNs (26,27) and TALENs (38) are either not robust enough or technically too complex to be routinely used to assess designer nuclease related off-target cleavage activity. Importantly, the published reports have shown that ZFN and RGN-driven off-target cleavage is largely based on sequence identity to the intended target site. Considering that context-dependent effects between the repeat units have not been reported for TALE-based DNA binding domains, it is reasonable to assume that TALEN binding to off-target sites also depends on sequence identity. Because of the lack of a biological assay, bioinformatics prediction is the only available system to predict potential off-target cleavage sites of TALENs. Given the potential of TALEN-mediated genome engineering in a therapeutic context, a more exhaustive analysis to relate nuclease-associated activity and toxicity with nuclease specificity is highly warranted.

Here, we have characterized the activity and toxicity of TALENs targeted to three different human loci. We show that our optimized TALEN scaffold (36) enables the generation of functional nuclease pairs that match the activity set by benchmark ZFNs. Importantly, our study revealed that TALEN expression in cell lines is associated with a low cytotoxicity. This observation was consistent with the absence of cell cycle aberrations and few genomic rearrangements as assessed at the *CCR2*/*CCR5* loci. Moreover, our results suggest that the benign cytotoxicity profile is due to a high specificity of TALENs, as evident from the low level of cleavage activity at predicted off-target sites. Hence, our data link low cytotoxicity to high specificity and establish the TALEN technology as a promising candidate for future clinical translation.

## MATERIALS AND METHODS

### Nuclease expression constructs

Target sites at the three chosen human loci where identified in proximity of benchmark ZFN targets without using previously published algorithms. TALE-based DNA binding domains were assembled using a previously described Golden Gate assembly kit (39) that was modified with four different Level 3 destination vectors to express functional nuclease monomers based on our previously optimized TALEN scaffold (36) (Δ135/+17; Supplementary Figure S1). These vectors included the 17.5th repeat and the wild-type *Fok*I cleavage domain (pVAX_CMV_TALshuttle(xx); ‘xx’ stands for the four different 17.5th RVDs used, NI, NG, HD and NN). The ZFN expression plasmids were generated by subcloning previously published zinc-finger arrays (codon-optimized and synthesized by GeneArt/Life Technologies, Regensburg) into the pRK5.N backbone (40), which includes an N-terminal HA tag and the SV40 nuclear localization domain and either of the obligate heterodimeric *Fok*I variant KV or EA (41) at the C-terminus. Amino acid sequences of the used ZFNs and TALENs are indicated in Supplementary Figure S1. The toxic ZFN (GZF1N/GZF3N) (40) was included as a positive control.

### Cell culture and transfection

HEK293T and HeLa FUCCI cells were cultured in Dulbecco's modified Eagle's medium (DMEM, PAA) supplemented with 10% Fetal Bovine Serum (FBS, PAA), 100 U/ml penicillin (PAA) and 100 μg/ml streptomycin (PAA). K562 cells were cultured in RPMI1640 media (Invitrogen) supplemented with 10% FBS, 100 U/ml penicillin and 100 μg/ml streptomycin, 2 mM L-glutamine (PAA). Primary human newborn foreskin fibroblasts (NuFFs) were maintained in low-glucose DMEM supplemented with 10% FBS, 100 U/ml penicillin and 100 μg/ml streptomycin, 2 mM L-glutamine and 1% Na-pyruvate (PAA). HEK293T and HeLa FUCCI cells were transfected using polyethylenimin (PEI), as described before (36). NuFF and K562 cells were nucleofected using the NHDF or V kit (Lonza) and programs U-023 and T-016, respectively, following the manufacturer instructions.

### Gene disruption

HEK293T and NuFF cells were transfected with 400 ng or 1.25 μg of each nuclease expression plasmid and pUC118 to normalize for DNA amount, harvested at the indicated time points to extract DNA using QIAamp DNA mini kit (Qiagen), the target loci amplified by polymerase chain reaction (PCR; see Supplementary Table S7 for primers), and then the extent of gene disruption assayed by T7 endonuclease 1 (T7E1, New England Biolabs) as previously described (36). For analysis under hypothermic conditions, cells were shifted to 30° for 3 days 4 h after transfection.

### Nuclease expression

HEK293T cells were PEI transfected, as described above, and harvested at indicated time points. Protein lysates were prepared using RIPA buffer (50 mM Tris, 150 mM NaCl, 1% NP-40, 0.1% SDS, 0.5% deoxycholate). ZFNs/TALENs or β-actin were detected using anti-HA tag (1:2000; Novus Biologicals) or anti-β-actin (1:2000; Cell Signaling) antibodies, respectively. Detection was performed using Horseradish Peroxidase (HRP)-conjugated anti-rabbit antibody (Dianova) and West Pico chemiluminescence substrate (Thermo Scientific).

### Gene expression

Total RNA was extracted from 1 × 10^6^ cells (HEK293T, NuFF or K562) using the TRIzol Reagent (Life Technologies) and reverse-transcribed with QuantiTect Reverse Transcription Kit (Qiagen). RT-PCR amplicons of *CCR5*, *AAVS1*, *IL2RG* and *GAPDH* loci were generated with primers listed in Supplementary Table S7 using program [1 min at 95°; 30 cycles of 95° for 5 s, 60° for 10 s and 72° for 7 s; 1 min at 72°] and resolved by 2% agarose gel electrophoresis.

### Nuclease-associated toxicity

Survival of HEK293T cells was assayed by measuring the decrease of mCherry-positive cells from day 2 to day 5, normalized to cells transfected with a non-functional nuclease expression vector as previously described (36). Cell cycle progression analysis in HeLa FUCCI was performed by seeding 100 000 cells 24 h prior PEI transfection with 1.6 μg of each nuclease monomer expression plasmid and 1.8 μg of pUC118. The fractions of cells in the respective cell cycle phase were determined by flow cytometry (FACSCalibur; BD Biosciences) 3 days post-transfection. Apoptosis was measured by staining HeLa FUCCI cells with an Allophycocyanin (APC)-conjugated AnnexinV antibody (BD Biosciences) according to the manufacturer's protocol 1 day post-transfection. 100 000 HEK293T cells were transfected as described above with increasing amounts (100–600 ng) of each nuclease monomer expression plasmid and stained for AnnexinV 3 days post-transfection. 100 ng of a mCherry expression vector was included to normalize for transfection efficiency and pUC118 up to 1.2 μg was added in each sample. Cell viability was measured by staining HEK293T with 7-aminoactinomycin D (7-AAD) 1 day post-transfection with nuclease expression vectors to allow the identification of dead cells by flow cytometry.

### Genomic rearrangements

Genomic DNA was extracted from HEK293T cells treated with ZFNs or TALENs using QIAamp DNA mini kit (Qiagen) and was subjected to PCR amplification using the diagnostic primers #1474 and #986 to detect deletions (amplicon length 477 bp) or #1474 and #985 to detect inversions (520 bp). PCR amplicons were resolved by 2% agarose gel electrophoresis. The percentage of Del/Inv as shown in Figure 5C was calculated as follows: the band intensities were estimated using Quantity One v.4.6.9 software (Biorad) and the amount of Del/Inv (middle band) was computed as percentage of total *CCR5* and *CCR2* alleles (upper and lower bands).

**Figure 1.**
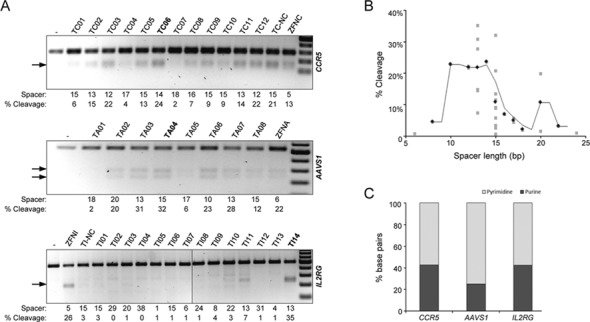
Assessment of TALEN activity at three human loci. (**A**) Disruption of target loci. HEK293T cells were transfected with expression plasmids encoding the indicated nuclease pair. Genomic DNA was extracted 3 days after transfection and subjected to PCR amplification of the target site followed by T7E1 assay. The spacer lengths between each nuclease half-site and the extent of cleavage (average percentage of modified alleles) are indicated below each panel. Arrows indicate the position of the expected cleavage products. The most active TALEN pairs are highlighted in bold. (**B**) Relation between spacer and TALEN activity. The plot shows the relationship between activity and the length of the DNA spacers separating the two TALEN half-sites. Each gray dot represents the average of three independent cleavage experiments (as shown in panel (A)), while the black dots embody the average values of all gray dots for a particular spacer length. A trend line connects the black dots. (**C**) Target site nucleotide composition. The histogram indicates the percentage of purines or pyrimidines in the target sites.

**Figure 2.**
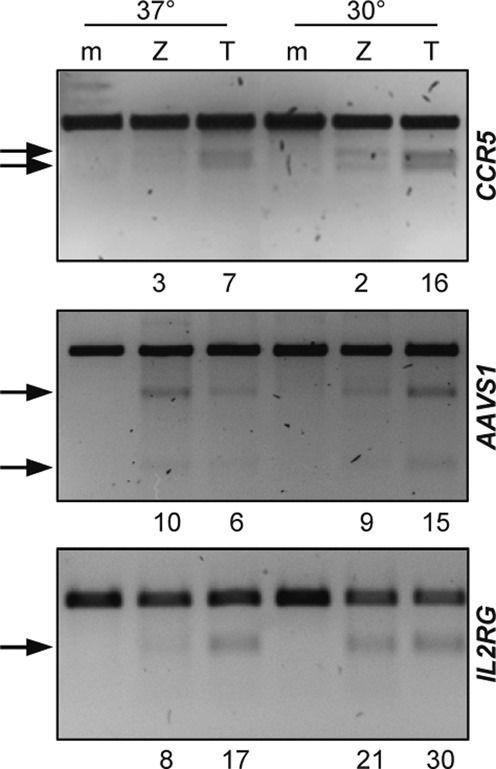
Designer nuclease activity in primary cells under normothermic and hypothermic conditions. Disruption of three different target loci was assayed in primary newborn human foreskin fibroblasts. Upon nucleofection with plasmids expressing the respective ZFNs or TALENs (TC06 for *CCR5*, TA04 for *AAVS1* and TI14 for *IL2RG*), cells were incubated at 37°C or 30°C for 3 days. Genomic DNA was extracted and the target sites amplified by PCR prior to digestion with T7E1. The arrows indicate the positions of the expected digestion products. The average percentage of modified alleles is indicated below each panel. m, mock; Z, ZFN; T, TALEN.

**Figure 3.**
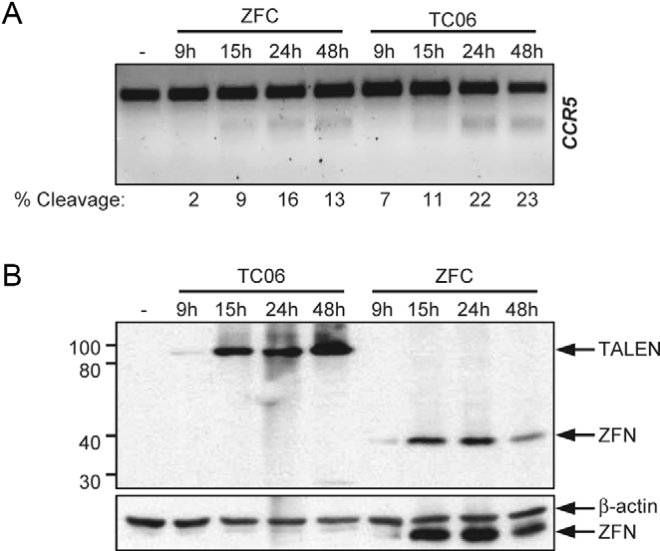
Kinetics of ZFN and TALEN expression and activity. HEK293T cells were transfected with the indicated *CCR5*-specific nuclease pairs and harvested at different time points after treatment. (**A**) Kinetics of nuclease activity. Target locus disruption was assayed by T7E1 assay and the extent of cleavage (as percentage of modified alleles) is indicated below each lane. (**B**) Kinetics of nuclease expression. Expression levels were determined by immunoblotting using antibodies against HA-tag (top panel) and β-actin (lower panel). Positions of TALENs, ZFNs and β-actin are indicated on the right and protein marker (in kDa) on the left.

**Figure 4.**
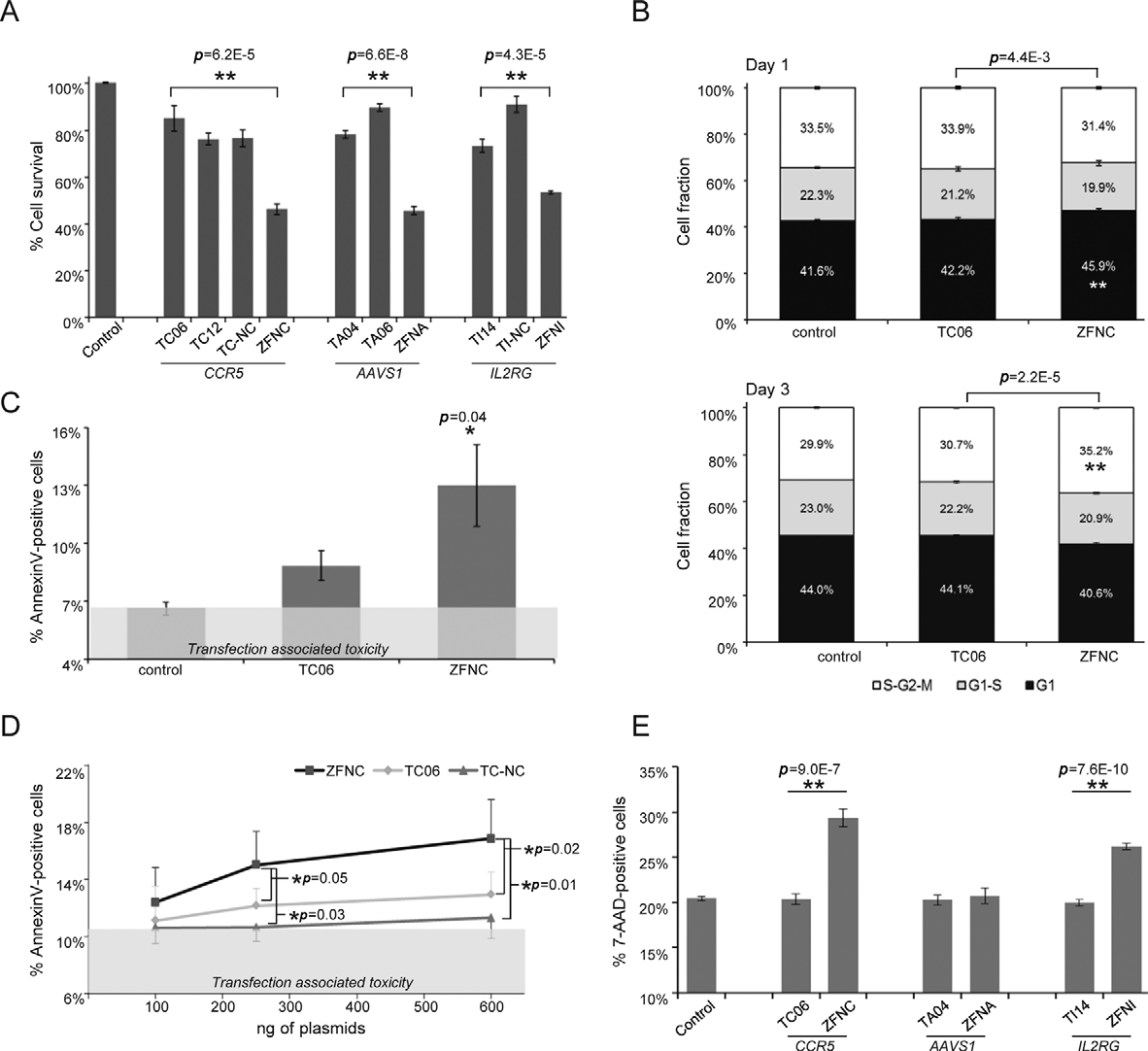
Nuclease-associated toxicity. (**A**) Cell survival. HEK293T cells were co-transfected with plasmids encoding the most active TALENs or ZFNs for the three loci tested together with a mCherry expression plasmid. The graph shows the fraction of mCherry-positive cells at day 5 as compared to day 2 post-transfection, relative to samples transfected with a control plasmid. (**B**) Cell cycle analysis. HeLa FUCCI cells were transfected to express the indicated nucleases and cell cycle progression was monitored by flow cytometry one (upper panel) and three (lower panel) days post-transfection. The graph shows the average percentage of cells in the respective cell cycle phase. (**C** and **D**) Nuclease-associated apoptosis. Apoptotic response to nuclease treatment was monitored by AnnexinV staining in HeLa FUCCI cells (C) or in HEK293T cells (D) transfected with increasing amounts of nuclease expression plasmids. The gray shaded areas indicate transfection-associated apoptosis. (**E**) Cell viability. HEK293T cells were transfected with expression vectors encoding the most active TALEN or ZFN pairs. The diagram shows the fraction of 7-AAD positive cells 1 day post-transfection. Statistically significant differences between ZFNs and controls or ZFNs and TALENs are indicated with an asterisk and the corresponding *p*-values (two-tailed, Student's t-test). Error bars indicate S.E.M.

**Figure 5.**
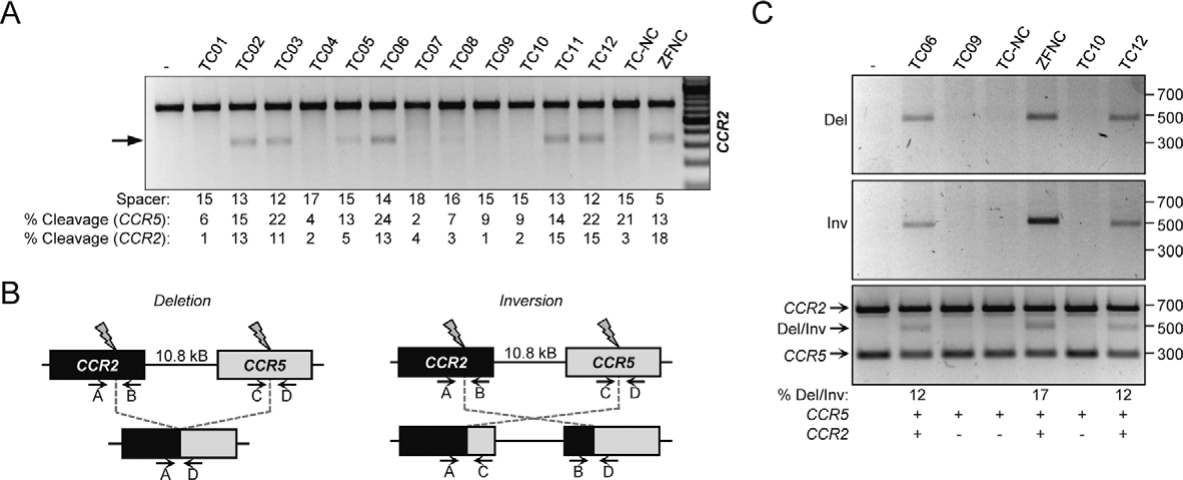
Nuclease-associated genotoxicity. (**A**) Off-target activity of *CCR5*-specific nucleases. The activity of the indicated nucleases at the human locus *CCR2* was monitored by T7E1. The spacer between the target half-sites and the average extent of cleavage at both *CCR5* and *CCR2* loci are indicated below. Arrow indicates the position of the expected digestion products. (**B**) Schematic of chromosomal organization of the *CCR2* and *CCR5* loci. The predicted genomic rearrangements that can occur upon simultaneous cleavage in both loci are shown: deletion (left panel) or inversion (right panel). Capital letters indicate positions of the primers used for molecular characterization. (**C**) Genomic rearrangements upon expression of designer nucleases. Genomic rearrangements were detected by PCR using the indicated primer pairs: A+D, deletion (Del, 477 bp, top); A+C, inversion (Inv, 520 bp, middle); A+B, *CCR2* allele (674 bp, bottom); C+D, *CCR5* allele (300 bp, bottom). Nuclease specificities and estimated percentage of Del/Inv products are indicated on the bottom. DNA-marker (in bp) is shown on the right.

### Off-target analysis

Potential off-target sites for the TALENs were chosen from the PROGNOS RVD-5TC and Homology-5TC rankings to contain a mixture of the top-ranked sites from both algorithms. In addition, *CCR2* was also interrogated for the TC-NC TALENs to facilitate comparison at that off-target site, even though this site did not appear in the 5TC PROGNOS rankings because of the lack of the 5′ pyrimidine in one of the half-sites. The different PROGNOS ranking algorithms employ various models of nuclease-DNA binding and cleavage preferences, including RVD association frequencies (42), the energy compensation model of cleavage (27) and the preference of ZFNs for guanosine residues (43). Genomic DNA was extracted from HEK293 cells 3 days post-transfection with ZFNC, ZFNA, TC06, TC-NC, TA04 or empty vector, as described above. PCR primers designed by PROGNOS (Supplementary Table S7) were used to amplify the predicted off-target loci and amplicons were sequenced using the RS SMRT technology (Pacific Biosciences). The results were analyzed by custom Perl scripts to identify reads containing evidence of NHEJ. The ‘specificity factor’ in Tables 1 and 2 is calculated as the ratio of on-target to off-target mutagenesis frequency based on the detected indels events shown in Supplementary Table S5.

**Table 1.** Off-target sites of *CCR5*-specific designer nucleases

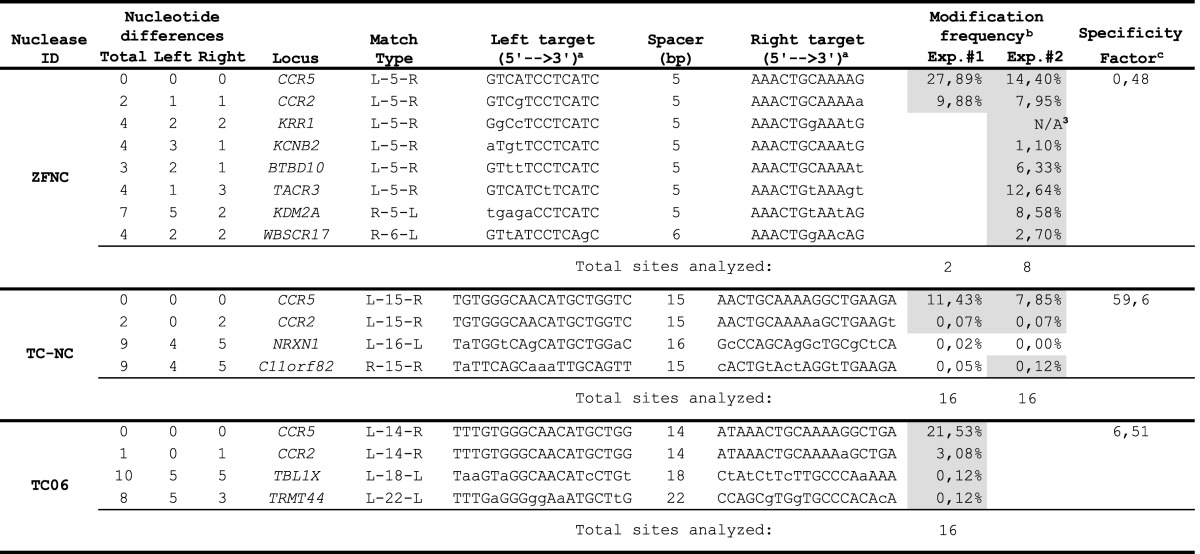											

^1^Nucleotide sequence differences as compared to the *CCR5* target site are indicated by lower-case letters.

^2^Sites with statistically significant enrichment of mutated sequences (*p* < 0.05) are highlighted by gray background.

^3^For this site the PCR amplification step was not successful.

^4^Ratio of the total on- to off-target modification frequencies.

**Table 2.** Off-target sites of *AAVS1*-specific designer nucleases

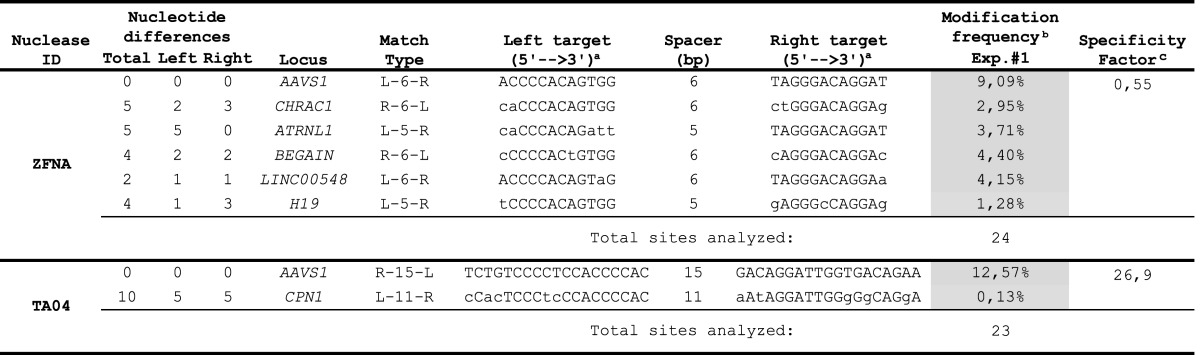										

^1^Nucleotide sequence differences as compared to the *AAVS1* target site are indicated by lower-case letters.

^2^Sites with statistically significant enrichment of mutated sequences (*p* < 0.05) are highlighted by gray background.

^3^Ratio of the total on- to off-target modification frequencies.

### Statistical analysis

Statistical differences reported in the nuclease-associated toxicity experiments represent the average of three independent experiments. Error bars indicate standard error of mean (S.E.M.). Statistical significance was determined using a two-tailed, homoscedastic Student's t-test. For off-target site analysis, the *p*-values were calculated for the one-sided alternative hypothesis that the modification frequency at the analyzed site (insertion/deletion) is greater for the nuclease treated cells compared to cells transfected with the empty vector.

## RESULTS

### Generation of highly active TALENs targeted to three human genes

We generated three sets of TALEN pairs targeted to the human loci *AAVS1, CCR5* and *IL2RG*, for which effective benchmark ZFNs are available (18,21,24). To this end, TALE DNA binding domains assembled with the ‘Golden Gate’ platform (39) were cloned in a TALEN expression plasmid containing an optimal architecture previously characterized in our lab (Scaffold A4-NH; (36)) (Supplementary Figure S1). A total of six nuclease monomers targeted to *AAVS1*, seven monomers targeting *CCR5* and eight monomers targeting *IL2RG* were produced and expressed at similar levels (Supplementary Figure S2). Differential pairing of these TALEN monomers allowed us to reconfirm the optimal spacer length of 12–15 bp between the TALEN target half-sites by analyzing the activity of 34 TALEN pairs termed TC#, TA# or TI#, targeting *CCR5*, *AAVS1* or *IL2RG*, respectively (Figure 1A and B, Supplementary Table S1**)**. In addition, previously validated TALENs targeted to *CCR5* (TC-NC) and *IL2RG* (TI-NC) based on a scaffold that includes a longer linker (+47 residues) were used as controls (36). Designer nuclease activity was assessed by measuring the extent of NHEJ-mediated mutagenic repair at the three target loci using the mismatch-sensitive T7 endonuclease 1 (T7E1) assay (Figure 1A). When tested in cultured cells, 19 out of 36 different TALEN pairs showed substantial activity, resulting in >5% of mutated target alleles. Considering only target sites with optimal spacers of 12–15 bp or 20–22 bp lengths, respectively, the success rate of producing active TALEN pairs ranged from 33% for the *IL2RG* locus to up to 90% and 100% for the *CCR5* and *AAVS1* loci, respectively. Notably, for all three loci we were able to identify at least one TALEN pair that performed similarly well as the respective benchmark ZFN, confirming the robustness of the chosen TALEN scaffold. Furthermore, our data show that 2 out of 20 TALEN monomers analyzed (L-169 and R-170) probably failed to bind to their respective target sites because they did not support cleavage in any of the tested nuclease configurations (Figure 1A). Thus, the success rate of generating functional monomers was ∼90%.

Assessment of the nucleotide composition of the 20 TALEN target half-sites (Figure 1C and Supplementary Figure S3) revealed that TALENs efficiently disrupted pyrimidine-rich target sites (*AAVS1*) as well as targets without any prevalence of certain nucleotides (*CCR5*). However, only two out of seven *IL2RG*-specific TALEN pairs, which fulfilled the spacer requirements, showed robust cleavage activity. The low targeting efficiency at this locus cannot be explained by the nucleotide sequence of the respective targets as the target site compositions in *IL2RG* are similar to those in *CCR5*. To explain the low success rate in generating functional TALEN pairs targeted to *IL2RG*, we assayed nuclease activity in various cell lines that revealed differential transcriptional activity of the target loci, as measured by semi-quantitative RT-PCR (Supplementary Figure S4A). Although *CCR5* was not expressed in HEK293T cells, it could be targeted with similar efficiencies as the ubiquitously expressed *AAVS1* locus (Figure 1A). Vice versa, we could not detect any improvement in TALEN activity at the *IL2RG* locus in K562 cells, in which *IL2RG* is expressed as opposed to HEK293T cells (Supplementary Figure S4B). These results suggest that the spacer length represents a major determinant for TALEN activity and that gene disruption efficiency seems not to be appreciably dependent on the transcriptional status of the target locus. However, the presence of CpG dinucleotides in TALEN target sites may negatively impact on the overall efficacy of TALEN monomers to bind to their intended targets, as shown recently (33,44,45). The majority of the *IL2RG*-specific ‘left’ TALEN monomers generated in this study are targeted to DNA sequences containing two or three CpG dinucleotides (Supplementary Table S1), which may explain the low success in generating functional *IL2RG*-specific TALENs. Indeed, the sole highly active *IL2RG*-specific TALEN pair consists of TALEN monomers targeted to CpG-free DNA sequences (Figure 1A and Supplementary Table S1; see nuclease pair TI14).

We next evaluated whether ZFNs and TALENs perform similarly well in a primary human cell type. Gene disruption frequencies of the most active TALEN pairs (TC06, TA04 and TI14) and our benchmark ZFNs were analyzed in primary human NuFFs. At all three loci, TALENs were similarly efficacious as the ZFNs, with allelic mutation frequencies ranging from 6% to 17% (Figure 2). The lower overall activity as compared to cell lines may be due to the lower transfection efficiency. However, the gene disruption activities of all nucleases were increased when cells were transiently incubated under hypothermic conditions (46).

To further characterize the two designer nuclease platforms, we profiled the kinetics of expression and activity of our *CCR5*-specific TALENs and ZFNs in HEK293T cells. The T7E1 assay revealed a comparable kinetics of activity for both platforms, with a plateau reached about 24 h post-transfection (Figure 3A). In terms of expression, the TALEN levels increased up to 48 h post-transfection while ZFNs reached a peak of expression 15 h after transfection (Figure 3B), which can be attributed to differences in protein stability and/or ZFN-associated cytotoxicity.

### Nuclease-associated cytotoxicity

Based on the findings that both nuclease activity and expression peak within 48 h after transfection, we assessed general cytotoxicity by measuring cell survival 5 days post-transfection. All tested TALENs outperformed ZFNs with an average of ∼80% of surviving cells expressing TALENs versus ∼50% for ZFN-expressing cells (Figure 4A). To study this effect in more detail, we investigated the impact of nuclease expression on cell cycle progression. We reasoned that nuclease-associated toxicity is probably caused by cleavage at off-target sites, which in turn arrests the cell cycle until the DSBs are repaired. To this end, we expressed the most active *CCR5*-specific TALEN TC06 and ZFNC in HeLa FUCCI cells that express fluorescently tagged cell cycle indicators (47). Flow cytometric cell cycle analysis after 1 and 3 days revealed that cells expressing TALENs were not different from control samples, while ZFN expression induced a significant accumulation of cells in G1 phase 1 day post-transfection followed by an accumulation in S-G2-M phase 3 days after transfection (Figure 4B). In agreement, the transient cell cycle deregulation was associated with a significant increase in apoptotic cells (Figure 4C), suggesting that a significant subset of cells was not able to repair the nuclease-induced DSBs, which in turn induced cell death. To correlate induction of apoptosis with nuclease expression, we transfected HEK293T cells with increasing amounts of *CCR5*-specific nuclease expression vectors. Augmenting the ZFNC load resulted in higher numbers of AnnexinV-positive cells (Figure 4D and Supplementary Figure S5). At medium and high concentrations, only the *CCR5*-specific ZFNs—but not TALENs TC06 and TC-NC—induced a significant level of apoptosis above background levels (note that experiments in Figure 4A and B have been performed using 400 ng of each nuclease plasmid and that TALEN and ZFN plasmids have a comparable size). Cells transfected with a previously described toxic ZFN pair (40) showed marked apoptosis in up to 32% of the total cell population (Supplementary Figure S5A). To further inspect nuclease-associated toxicity, we stained HEK293T cells with 7-AAD 2 days post-transfection with expression vectors encoding the most active nuclease for each of the three loci. This chemical compound intercalates in the DNA double helix with high affinity. However, since 7-AAD cannot penetrate an intact cell membrane, it is used to effectively discriminate between live and dead cells. While samples expressing TALENs did not show any difference compared to control samples, expression of ZFNs targeted to *CCR5* and *IL2RG* led to a significant increase in 7-AAD positive cells (Figure 4E). In summary, expression of TALENs was well tolerated by human cells.

### Specificity of designer nucleases targeted to *CCR5* and *AAVS1* loci

As the *CCR5*-specific ZFNs are the only designer nuclease pair in clinical trials, they are the best-characterized nucleases in terms of specificity to date (26,27). On the other hand, *AAVS1*-specific ZFNs are broadly used in the genome engineering field because the locus is considered a ‘safe harbor’ (48) but only few specificity data are available for this ZFN (21). We therefore profiled off-target activity of our *CCR5-* and *AAVS1*-specific ZFNs and TALENs. First, we investigated the activity of the *CCR5*-specific nucleases at the *CCR2* locus that has been reported to be a prominent off-target site of *CCR5*-specific ZFNs (26,27,49). *CCR2* is located about 15 kb upstream of the *CCR5* locus on chromosome 3 and its coding sequence is highly similar to *CCR5*. Within a 60-bp stretch that surrounds the identified ZFN off-target site in *CCR2*, only five nucleotides are different from the corresponding stretch in the *CCR5* locus (Supplementary Table S2). Gene disruption at the *CCR2* locus was evaluated for the entire panel of *CCR5*-specific TALEN pairs and the benchmark ZFN by the T7E1 assay in HEK293T cells (Figure 5A). While ZFNC showed considerable activity at *CCR2*, some of the *CCR5*-specific TALENs revealed little or no off-target activity at this locus. Importantly, TALEN pair TC-NC showed high on-target mutagenic activity at *CCR5* but undetectable off-target cleavage at *CCR2*, demonstrating that rational design may allow for the generation of TALEN pairs that can discriminate between highly identical targets in the human genome.

A potential deleterious effect driven by the generation of two simultaneous DSBs in a genome is the induction of genomic rearrangements. To investigate whether concomitant cleavage at *CCR5* and *CCR2* induces deletions or inversion of large genomic fragments on chromosome 3, as previously shown (49,50), we genotyped a subset of samples transfected with *CCR5*-specific nucleases by qualitative PCR (Figure 5B). We monitored deletions as well as inversions occurring between *CCR2* and *CCR5* in samples treated with the ZFN pair and TALENs TC06 and TC12 that were unable to discriminate between the two loci (Figure 5C). However, chromosomal rearrangements were not detected in samples treated with TALEN pairs active only at *CCR5* (TC09, TC10, TC-NC). To estimate the extent of rearranged alleles in the transfected cell population, we mixed the four diagnostic primers in a single PCR reaction and computed the frequency of deletions or inversions (Del/Inv) for the non-discriminating nucleases (ZFNC, TC06, TC12) to be in the range of about 15% of the total number of alleles. The TALEN pair TC-NC did not induce detectable levels of genomic rearrangement between the two loci, emphasizing the importance of minimizing off-target cleavage, in particular on the same chromosome.

We next investigated the specificity of our *CCR5-* and *AAVS1*-specific designer nucleases by high-throughput sequencing at potential off-target sites predicted *in silico* using the recently developed PROGNOS software (http://bit.ly/PROGNOS). When applying the bioinformatics tool, four of seven previously validated off-target sites (Table 1; (26,27)) were listed in the top 32 off-target sites for the *CCR5*-specific ZFN (Supplementary Table S3). For the *AAVS1*-specific ZFN, few specificity data are available in the literature. PROGNOS confirmed the prediction of two out of five previously identified but not validated (21) off-target sites in the top 23 list of predicted off-target sites (Supplementary Table S3). We also used PROGNOS to predict the top 15 off-target sites for TC06 and TC-NC, respectively, and the top 22 off-target sites for the *AAVS1*-specific TALEN TA04.

For the *CCR5*-specific ZFN, we confined high-throughput sequencing analysis to those sites that were previously identified and we confirmed off-target cleavage activity at six out of seven sites analyzed, sometimes with disruption activities over 12%, i.e. almost as high as at the *CCR5* locus. Moreover, PROGNOS was able to predict a novel off-target site on chromosome 5 (51). Analysis of off-target cleavage of the *AAVS1*-specific ZFN pair revealed activity of up to 4.4% at 5 out of the 23 predicted off-target sites (Table 2 and Supplementary Table S5). Hence, we validated three previously predicted off-target sites and identified and validated two additional off-target sites for the *AAVS1*-specific ZFN. In addition to *CCR2*, the two *CCR5*-specific TALEN pairs exhibited minor off-target cleavage activity (0.12%) at a total of three off-target sites. The *AAVS1*-specific TALEN pair revealed statistically significant activity at 1 (0.13%) out of the 22 predicted off-target sites (Table 2 and Supplementary Table S5).

The majority of mutations retrieved at the analyzed loci consisted of short deletions in the respective spacer sequences, as previously reported (52), and DNA repair seemed to be driven mostly by microhomology-mediated repair (Supplementary Table S6 and data not shown).

In summary, the high-throughput sequencing results exposed important information regarding the specificity of the assessed nucleases. In particular, compared to the benchmark ZFN, our *CCR5*-specific TALENs are better tolerated when expressed in human cells and can be designed to discriminate between highly identical sequences, such as *CCR5* and *CCR2*. The *CCR5*:*CCR2* targeting ratio was determined to be in the range of 3:1 for the ZFN, and 130:1 or 7:1 for the TALEN pairs TC-NC and TC06, respectively. The calculated ‘specificity factor’, indicating the ratio of the frequency of on-target mutagenesis to the overall frequency of off-target mutagenesis, was 1:2 for the *CCR5*-specific ZFN, 60:1 for TALEN TC-NC and 7:1 for TALEN TC06 (Table 1). A similar trend was observed for the *AAVS1*-specific nucleases. While we identified and validated five novel off-target sites for the *AAVS1*-specific benchmark ZFN, with a specificity factor of 1:2, we detected off-target cleavage activity at a single predicted site for TALEN TA04, with a calculated specificity factor of 27:1 (Table 2).

## DISCUSSION

Targeted genome editing with engineered nucleases has been successfully applied in various organisms and cell types (1,3,15). However, a study that correlates activity, cytotoxicity and specificity highlighting advantages and limitations associated with the use of the various nuclease platforms is still lacking. Here we provide the first thorough characterization of a panel of designer TALENs targeted to three human genes for which effective benchmark ZFNs are available (18,21,24). We identified TALENs that show activity comparable to the matching ZFNs both in human cell lines and in primary human fibroblasts. Assessment of nuclease-associated toxicity in human cells revealed that overexpression of TALENs is better tolerated than that of benchmark ZFNs. Moreover, profiling nuclease specificity in HEK293T confirmed off-target activity of the *CCR5*-specific ZFNs and provides the first data for the *AAVS1*-specific ZFN pair with five novel off-target sites identified. In contrast, the best performing *CCR5-* and *AAVS1*-specific TALEN pairs showed only negligible activity at a total of 49 off-target sites analyzed.

Using our optimized TALEN scaffold that includes a 17 residue linker (+17aa) between the last TALE repeat and the *Fok*I endonuclease domain (36), we were able to generate effective nucleases able to induce modifications in up to 30% of alleles at three human loci, which was similarly high as the benchmark ZFNs. In line with previous reports (13,14), we achieved a high success rate in generating functional TALEN monomers, regardless of the nucleotide composition of the target sequence and without respecting any previously published design guidelines. Furthermore, we corroborate that our TALEN scaffold with a short +17 residue linker works optimally on DNA spacers of 10–15 bp, with a second peak of activity on 20–22 bp long spacers. This second peak of activity is consistent with formation of an active TALEN complex spaced to include an additional DNA helix turn, and in line with observations previously made for ZFNs (53,54). Most of the 16 non-functional TALEN pairs tested hence failed to cleave the target DNA because the spacer length requirements were not fulfilled. Considering only target sites with optimal spacer lengths, the success rate of producing active TALEN pairs ranged from 33% for the *IL2RG* locus to 90% for the *CCR5* locus and 100% for the *AAVS1* locus.

The impact of the chromatin structure and/or the epigenetic status of a locus on nuclease activity is still poorly understood. The low success frequency at *IL2RG* could therefore be attributed to chromatin structure and/or DNA methylation that may negatively affect accessibility or DNA binding affinity of the TALENs (33). Our results suggest that the transcriptional status of a locus is not a major determinant of TALEN activity. Interestingly however, the presence of CpG dinucleotides within the target sequences seems to substantially affect TALEN activity. While all the *CCR5*- and *AAVS1*-specifc TALENs and the only highly active *IL2RG*-specific TALEN pair identified in this study bind to CpG-free DNA target sequences, the non-functional *IL2RG*-specific TALEN pairs contain two or three CpG dinucleotides in the left target subsite. A more systematic approach is needed to address this point. In line with a recent report in zebrafish (33) and with previously published data showing that the HD module is not able to bind to 5mC (45), our data emphasize that CpG dinucleotides should be avoided when searching for amenable TALEN target sites in the human genome. Intriguingly, activity of the *IL2RG*-specific ZFN pair was not affected by the presence of two CpG dinucleotides in the left target subsite. This observation raises important considerations related to the TALEN platform. The reduced activity of TALENs at sites containing CpG dinucleotides may decrease the potential target range in the human genome on the one hand (unless modules that allow binding to 5mC are used) but, on the other hand, increases specificity as potential off-target sites in methylated genome regions cannot be cleaved.

Nuclease specificity is a key factor for advancing targeted genome engineering into the clinic. Here we show that expression of TALENs is generally well tolerated by human cells. In contrast, expression of our ZFN pairs reduced cell survival, was associated with cell cycle arrest and increased cell death, all of which suggest surplus DNA cleavage at off-target sites. Using the newly developed PROGNOS bioinformatics tool (51), we predicted potential ZFN and TALEN off-target sites in the human genome and screened them by high-throughput sequencing. Importantly, the top 32 ranked potential off-target sites of the *CCR5*-specific ZFN included four off-target sites in the top seven that were previously verified experimentally (26,27) and one novel site (51). The top 23 predicted off-target sites for the *AAVS1*-specific ZFN included two previously predicted sites and allowed us to verify a total of five novel off-target sites. PROGNOS was also successfully employed to predict four novel off-target sites for TALENs targeting beta-globin (51). These results clearly validate and underline the high predictive value of PROGNOS that can be reliably applied to predict both ZFN and TALEN off-target sites.

In our study we have profiled TALEN off-target activity in a non-clonal cell population with high-throughput sequencing. Many previous reports have either focused on identifying the integration site of non-integrating viral vectors trapped in TALEN-induced DSBs (38) or using Surveyor/T7E1 assays to monitor off-target activity at genomic loci predicted by bioinformatics (55). However, the small number of off-target sites identified and analyzed or the use of genomic DNA extracted from clonally derived cell populations or live animals (56,57) may likely not provide enough depth to identify rare mutagenic events at off-target sites as those identified here. The five novel off-target sites of the widely used *AAVS1*-specific ZFN are certainly of importance to those researchers currently using or planning to use this nuclease pair for targeted integration at the *AAVS1* ‘safe harbor’ locus. Given the superior toxicity profile and the absence of significant off-target activity, our *AAVS1*-specific TALEN pair TA04 might be a valid alternative for targeted gene addition into the *AAVS1* ‘safe harbor’ site, in particular in primary cells.

Due to the high sequence similarity with *CCR5*, assessment of nuclease specificity confirmed that the major off-target sites of our *CCR5*-specific ZFN and TALEN pairs were found in the *CCR2* locus. As reported before, concomitant cleavage at *CCR2* and *CCR5* results in deletion of the intervening sequence (30,49). We show that nucleases, which are not specific enough to discriminate between these two highly similar loci (i.e. ZFNC and TC06), induce deleterious genomic rearrangements, including deletions and inversions. Even though not investigated further, we envision that simultaneous off-target activity on two different chromosomes can induce translocations between two major off-target sites, as previously shown (58). Importantly, we show that such deleterious genotoxic events can be restrained by using rationally designed nucleases, such as TALEN TC-NC, which shows an unprecedented *CCR5*:*CCR2* targeting ratio of ∼130:1. We speculate that the ability of TALEN TC-NC in discriminating between *CCR5* and *CCR2* is based on the fact that one of the two TC-NC subunits targets a T in position 0, which is not present in *CCR2*. Thus, the 5′-T can be used as a major discriminant between highly similar off-target sites to overcome cytotoxic side effects.

Examination of the few off-target sites shows that TALENs tolerate up to five mismatches in their 19-bp target half-sites. Whether the position of these mismatches has an impact on the overall binding affinity of TALEN monomers has not been addressed here. In any case, a systematic analysis of TALEN off-target activity may provide novel design guidelines that can be taken into consideration in the future to avoid potential off-target activity at sites with more than 74% nucleotide identity to the intended target site. Our sequencing data revealed some few off-target mutagenic events at predicted homodimeric TALEN off-target sites for our *CCR5*-specific TALENs. Thus, TALEN specificity can be further increased by coupling the TALE DNA binding domains to obligate heterodimeric *Fok*I endonuclease variants (59,60) that have been shown to abolish cleavage of ZFNs at homodimeric off-target sites (26). Even though most of our findings are based on TALENs with the Δ135/+17 scaffold developed in our lab (36), we believe that they can be extended to alternative TALEN designs with longer linkers (61). Importantly, as shown for ZFNs (53), our short 17-residue linker may improve specificity by limiting spacer tolerability. Additionally, intranuclear concentration of the designer nucleases and duration of the expression are important parameters affecting cleavage specificity. For instance, delivery of a *CCR5*-specific ZFN pair in the form of proteins resulted in short persistence in the transduced cells and was associated with reduced off-target activity at *CCR2* (62).

We systematically measured higher off-target activity of the *CCR5*-specific ZFNs than previously published (26,27). We reason that this is most likely due to the cell line (HEK293T versus K562 cells) and the transfection method (PEI versus nucleofection) that we used, which may result in higher intranuclear nuclease expression levels. Also, our ZFNs contain a slightly different obligate heterodimeric *Fok*I domain (41) compared to the most commonly used one (63) and a different epitope tag (HA versus FLAG). Given that we were able to identify previously reported off-target sites at much higher frequencies demonstrates that PEI-mediated transfection of HEK293T cells represents an ideal ‘mutation-prone system’ to evaluate nuclease specificity and further emphasizes the high specificity of the TALENs tested here.

Taken together, our results establish TALENs as a safe and specific platform to edit the human genome, paving the way for their use for clinical translation. As mentioned previously, RGNs have been established as a versatile tool to modify the human genome (15). Although there has been some concern regarding their specificity (28–30), it will be interesting to see whether this novel class of designer nucleases can match the high specificity set by TALENs, e.g. as recently shown by truncating the gRNA molecule (64).

## SUPPLEMENTARY DATA

Supplementary data are available at NAR Online.

SUPPLEMENTARY DATA
