# Supplementary material for: TALENs facilitate targeted genome editing in human cells with high specificity and low cytotoxicity
Source: Nucleic Acids Res. 2014 May 3;42(10):6762–73. doi: 10.1093/nar/gku305 (PMC4041469; doi:10.1093/nar/gku305)
Supplement: SUPPLEMENTARY DATA [file supp_42_10_6762__index.html]

TALENs facilitate targeted genome editing in human cells with high specificity and low cytotoxicity — TALENs facilitate targeted genome editing in human cells with high specificity and low cytotoxicity — SUPPLEMENTARY DATA 

# TALENs facilitate targeted genome editing in human cells with high specificity and low cytotoxicity

## SUPPLEMENTARY DATA

**Files in this Data Supplement:**

- SUPPLEMENTARY DATA
