# Supplementary material for: TALENs facilitate targeted genome editing in human cells with high specificity and low cytotoxicity
Source: Nucleic Acids Res. 2014 May 3;42(10):6762–73. doi: 10.1093/nar/gku305 (PMC4041469; doi:10.1093/nar/gku305)
Supplement: SUPPLEMENTARY DATA [file supp_gku305_nar-03711-h-2013-File007.pdf]

Supplementary material

TALENs mediate genome editing with superior specificity and lower toxicity than matched ZFNs

Mussolino C., Alzubi J., Fine E.J., Morbitzer R., Cradick T.J., Lahaye T., Bao G. and Cathomen T.

**Supplementary Figure S1**	Amino acid sequences of the TALEN scaffold and ZFNs used in this study
**Supplementary Figure S2**	TALEN and ZFN monomers expression levels
**Supplementary Figure S3**	Basepair composition of the 20 TALEN target half-sites
**Supplementary Figure S4**	Relationship between nuclease activity and target locus expression
**Supplementary Figure S5**	*CCR5* -specific designer nuclease associated toxicity
**Supplementary Table S1**	Target sequences of the nuclease pairs used in this study
**Supplementary Table S2**	Target sites in *CCR5* and *CCR2*
**Supplementary Table S3**	Off-target sites of *CCR5* - and *AAVS1* -specific zinc finger nucleases predicted by PROGNOS tool
**Supplementary Table S4**	Off-target sites of CCR5- and AAVS1-specific TALENs predicted by PROGNOS tool
**Supplementary Table S5**	Results of the SMRT sequencing
**Supplementary Table S6**	Mutations induced by *CCR5* -specific designer nucleases
**Supplementary Table S7**	List of primers used in this study

Mussolino *et al*. Supplementary Figure S1

a

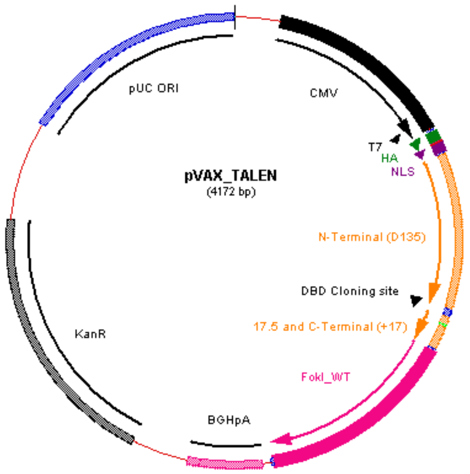

b

N-Terminal (Δ135):

APRRRAAQPSDASPAAQVDLRTLGYSQQQQEKIKPKVRSTVAQHHEALVGHGFTHAHIVALSQHPAALGTVAVKYQDM
IAALPEATHEAIVGVGKQWSGARALEALLTVAGELRGPPLQLDTGQLLKIAKRGGVTAVEAVHAWRNALTGAPLN

Aligned repeat domains (DBD):

1 LTP(e/q)QVVAIAS**xx**GGKQALETVQ(r/a)LLPVLCQAHG
2 LTP(e/q)QVVAIAS**xx**GGKQALETVQ(r/a)LLPVLCQAHG
3 LTP(e/q)QVVAIAS**xx**GGKQALETVQ(r/a)LLPVLCQAHG
4 LTP(e/q)QVVAIAS**xx**GGKQALETVQ(r/a)LLPVLCQAHG
5 LTP(e/q)QVVAIAS**xx**GGKQALETVQ(r/a)LLPVLCQAHG
6 LTP(e/q)QVVAIAS**xx**GGKQALETVQ(r/a)LLPVLCQAHG
7 LTP(e/q)QVVAIAS**xx**GGKQALETVQ(r/a)LLPVLCQAHG
8 LTP(e/q)QVVAIAS**xx**GGKQALETVQ(r/a)LLPVLCQAHG
9 LTP(e/q)QVVAIAS**xx**GGKQALETVQ(r/a)LLPVLCQAHG
10 LTP(e/q)QVVAIAS**xx**GGKQALETVQ(r/a)LLPVLCQAHG
11 LTP(e/q)QVVAIAS**xx**GGKQALETVQ(r/a)LLPVLCQAHG
12 LTP(e/q)QVVAIAS**xx**GGKQALETVQ(r/a)LLPVLCQAHG
13 LTP(e/q)QVVAIAS**xx**GGKQALETVQ(r/a)LLPVLCQAHG
14 LTP(e/q)QVVAIAS**xx**GGKQALETVQ(r/a)LLPVLCQAHG
15 LTP(e/q)QVVAIAS**xx**GGKQALETVQ(r/a)LLPVLCQAHG
16 LTP(e/q)QVVAIAS**xx**GGKQALETVQ(r/a)LLPVLCQAHG
17 LTP(e/q)QVVAIAS**xx**GGKQALETVQ(r/a)LLPVLCQAHG

17.5th repeat (underlined)and C-Terminal (+17):

LTPQQVVAIAS**xx**GGRPALESIVAQLSRPDPALAALT

RVDs (xx):

HD: CACGAC
NI: AATATC
NG: AATGGC
NN: AATAAT

c

>ZFNC_Left
MGYPYDVPDYASRPKKKRKVGIHASPAAMAERPFQCRICMRNFS**DRSNLSR**HIRTHTGEKPFACDICGRKFA**ISSNLN**
SHTKIHTGSQKPFQCRICMRNFS**RSDNLAR**HIRTHTGEKPFACDICGRKFA**TSGNLTR**HTKIHLRGSQLVKSELEEKK
SELRHKLKYVPHEYIELIEIARNSTQDRILEMKVMEFFMKVYGYRGKHLGGSRKPDGAIYTVGSPIDYGVIVDTKAYS
GGYNLPIGQADEMERYVEENQTRNKHANPNEWWKVYPSSVTEFKFLFVSGHFKGNYKAQLTRLNHITNCNGAVLSVEE
LLIGGEMIKAGTLTLEEVRRKFNNGEINF

>ZFNC_Right
MGYPYDVPDYASRPKKKRKVGIHASPAAMAERPFQCRICMRNFS**RSDNLSV**HIRTHTGEKPFACDICGRKFA**QKINLQ**
VHTKIHTGEKPFQCRICMRNFS**RSDVLSE**HIRTHTGEKPFACDICGRKFA**QRNHRTT**HTKIHLRGSQLVKSELEEKKS
ELRHKLKYVPHEYIELIEIARNSTQDRILEMKVMEFFMKVYGYRGKHLGGSRKPDGAIYTVGSPIDYGVIVDTKAYSG
GYNLPIGQADEMQRYVKENQTRNKHINPNEWWKVYPSSVTEFKFLFVSGHFKGNYKAQLTRLNHVTNCNGAVLSVEEL
LIGGEMIKAGTLTLEEVRRKFNNGEINF

>ZFNA_Left
MGYPYDVPDYASRPKKKRKVGIHASPAAMAERPFQCRICMRNFS**YNWHLQR**HIRTHTGEKPFACDICGRKFA**RSDHLT**
THTKIHTGSQKPFQCRICMRNFS**HNYARDC**HIRTHTGEKPFACDICGRKFA**QNSTRIG**HTKIHLRGSQLVKSELEEKK
SELRHKLKYVPHEYIELIEIARNSTQDRILEMKVMEFFMKVYGYRGKHLGGSRKPDGAIYTVGSPIDYGVIVDTKAYS
GGYNLPIGQADEMERYVEENQTRNKHANPNEWWKVYPSSVTEFKFLFVSGHFKGNYKAQLTRLNHITNCNGAVLSVEE
LLIGGEMIKAGTLTLEEVRRKFNNGEINF

>ZFNA_Right
MGYPYDVPDYASRPKKKRKVGIHGVPAAMAERPFQCRICMRNFS**QSSNLAR**HIRTHTGEKPFACDICGRKFA**RTDYLV**
DHTKIHTGSQKPFQCRICMRNFS**YNTHLTR**HIRTHTGEKPFACDICGRKFA**QGYNLAG**HTKIHLRGSQLVKSELEEKK
SELRHKLKYVPHEYIELIEIARNSTQDRILEMKVMEFFMKVYGYRGKHLGGSRKPDGAIYTVGSPIDYGVIVDTKAYS
GGYNLPIGQADEMQRYVKENQTRNKHINPNEWWKVYPSSVTEFKFLFVSGHFKGNYKAQLTRLNHVTNCNGAVLSVEE
LLIGGEMIKAGTLTLEEVRRKFNNGEINF

>ZFNI_Left
MGYPYDVPDYASRPKKKRKVGIHGVPAAMAERPFQCRICMRNFS**RSDTLSE**HIRTHTGEKPFACDICGRKFA**ARSTRT**
THTKIHTGSQKPFQCRICMRNFS**RSDSLSK**HIRTHTGEKPFACDICGRKFA**QRSNLKV**HTKIHLRGSQLVKSELEEKK
SELRHKLKYVPHEYIELIEIARNSTQDRILEMKVMEFFMKVYGYRGKHLGGSRKPDGAIYTVGSPIDYGVIVDTKAYS
GGYNLPIGQADEMERYVEENQTRNKHANPNEWWKVYPSSVTEFKFLFVSGHFKGNYKAQLTRLNHITNCNGAVLSVEE
LLIGGEMIKAGTLTLEEVRRKFNNGEINF

>ZFNI_Right
MGYPYDVPDYASRPKKKRKVGIHGVPAAMAERPFQCRICMRNFS**RSDNLSV**HIRTHTGEKPFACDICGRKFA**RNAHRI**
NHTKIHTGSQKPFQCRICMRNFS**RSDTLSE**HIRTHTGEKPFACDICGRKFA**ARSTRTN**HTKIHLRGSQLVKSELEEKK
SELRHKLKYVPHEYIELIEIARNSTQDRILEMKVMEFFMKVYGYRGKHLGGSRKPDGAIYTVGSPIDYGVIVDTKAYS
GGYNLPIGQADEMQRYVKENQTRNKHINPNEWWKVYPSSVTEFKFLFVSGHFKGNYKAQLTRLNHVTNCNGAVLSVEE
LLIGGEMIKAGTLTLEEVRRKFNNGEINF

Supplementary Figure S1. Amino acid sequences of the TALEN scaffold and ZFNs used in this study. (**a**) “Level 3” TALEN destination vector. The functional portions of the Level 3 TALEN destination vector include the CMV promoter, SV40 nuclear localization signal (NLS), HA-tag (HA) and WT *Fok*I cleavage domain (FokI_WT). The DNA binding domain (DBD) is cloned in the DBD cloning site. (**b**) Protein sequence of TALEN scaffold. The protein sequence of our previously published optimal TALEN architecture (Δ135/+17) is shown. In the central repeat domains, amino acid variability is shown in brackets (Reviewed in 6) and “xx” indicates the repeat variable di-residues (RVDs) defined below. (**c**) ZFNs protein sequences. The recognition α-helices are highlighted in bold, the obligate heterodimeric *Fok*I domains are underlined.

Mussolino *et al*. Supplementary Figure S2

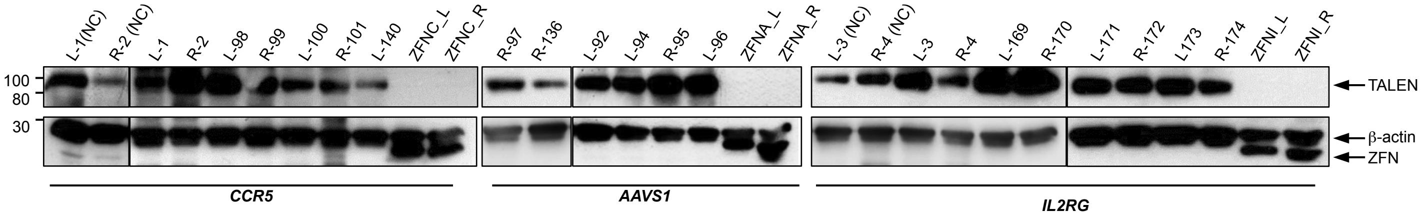

Supplementary Figure S2. TALEN and ZFN monomer expression levels. HEK293T cells were transfected with a single nuclease expression plasmid using PEI and harvested 48 hours later. Immunoblotting of equal amounts of cell lysates was performed with antibodies recognizing the HA-tag epitope (TALENs or ZFNs) and β-actin to monitor for protein load. The figure is a combination of different gel images, each separated by vertical black lines. The protein ladder is indicated on the left (kDa).

Mussolino *et al*. Supplementary Figure S3

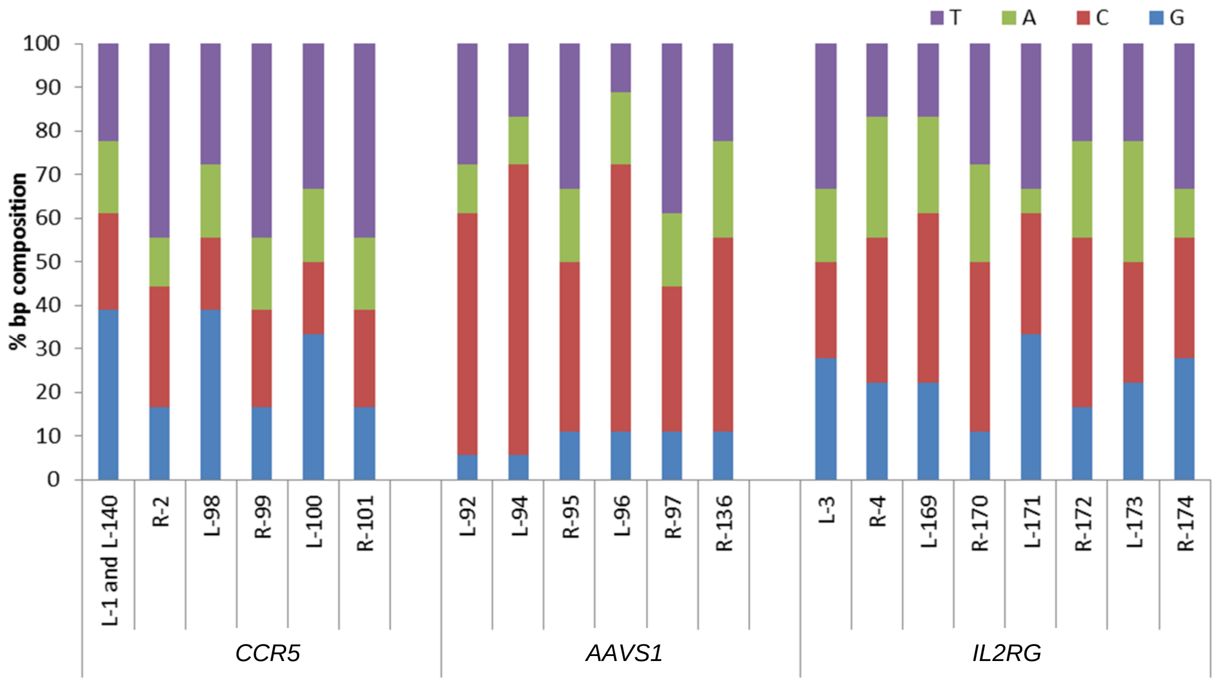

Supplementary Figure S3. Basepair composition of the 20 TALEN target half-sites. The graph depicts the nucleotide compositions of the 20 target sites in the three loci. Note that the *CCR5*-specific TALEN monomers L-1 and L-140 target the same site but were cloned into two different plasmid backbones (pAC and pVAX, respectively). The 5'-T in position 0 was not included.

Mussolino *et al*. Supplementary Figure S4

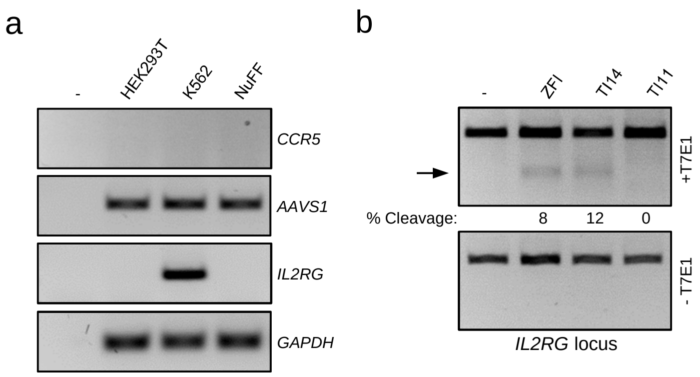

Supplementary Figure S4. Relationship between nuclease activity and target locus expression. (**a**) Expression profiles of the target loci in three different cell lines. Expression was determined by semi-quantitative RT-PCR. GAPDH was used as a control. (**b**) Disruption of the human *IL2RG* locus in K562 cells. Genomic DNA was extracted from cells 3 days post-nucleofection and subjected to PCR amplification of the target locus, followed by T7E1 assay (upper panel) or left untreated (lower panel). The arrow indicates the expected position of the digestion product. The average percentage of modified alleles is indicated.

Mussolino *et al*. Supplementary Figure S5

a

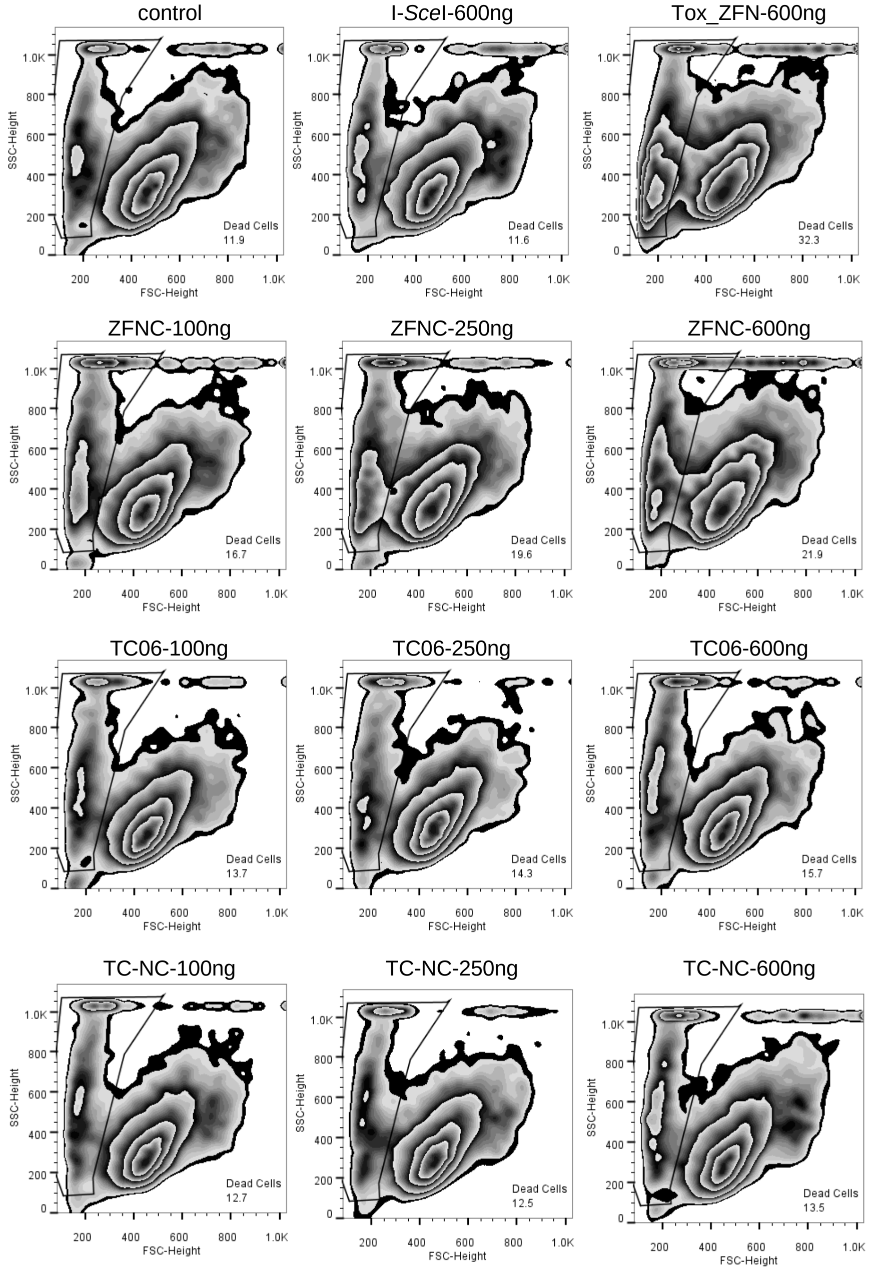

b

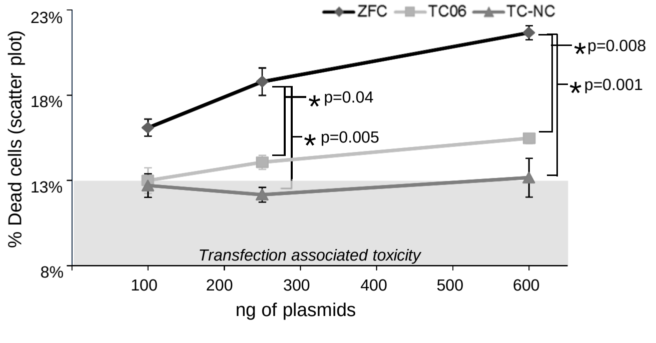

Supplementary Figure S5. *CCR5*-specific designer nuclease associated toxicity. (**a**) Assessment of cell death by scatter plots analysis. The scatter plots were used to determine the extent of dead cells upon transfection of designer nuclease expression vectors in HEK293T cells. The non-toxic meganuclease I-*Sce*I and a toxic ZFN pair (GZF1N/GZF3N) equipped with WT *Fok*I cleavage domain were used as controls. A representative experiment from each triplicate is shown. (**b**) Nuclease-associated toxicity. Correlation of dead cells (gated as shown in panel a) and the amounts of designer nucleases plasmids transfected. Statistically significant differences are indicated by an asterisk and the corresponding p-values, as calculated by the two tailed Student's t-test. The grey shaded area indicates the background due to transfection-associated toxicity. Data represent two independent experiments performed in triplicates. Error bars, S.E.M.

Supplementary Table S1. Target sequences of the nuclease pairs used in this study

Nuclease ID	Nuclease pair	Target Gene	Target sequence (5'…3')[1]	Spacer (bp)[2]	Cleavage (% ± SEM)[3]
TC01	L-1 + R-2	*CCR5*	5'-GGTTT**TGTGGGCAACATGCTGGTC**ATCCTCATCCTGATA**AACTGCAAAAGGCTGAAGA**GC-3'	15	5.7% ± 0.8%
TC02	L-1 + R-99	*CCR5*	5'-GGTTT**TGTGGGCAACATGCTGGTC**ATCCTCATCCTGA**TAAACTGCAAAAGGCTGAA**GAGC-3'	13	14.8% ± 2.1%
TC03	L-1 + R-101	*CCR5*	5'-GGTTT**TGTGGGCAACATGCTGGTC**ATCCTCATCCTG**ATAAACTGCAAAAGGCTGA**AGAGC-3'	12	21.7% ± 2.0%
TC04	L-98 + R-2	*CCR5*	5'-GGT**TTTGTGGGCAACATGCTGG**TCATCCTCATCCTGATA**AACTGCAAAAGGCTGAAGA**GC-3'	17	4.4% ± 0.7%
TC05	L-98 + R-99	*CCR5*	5'-GGT**TTTGTGGGCAACATGCTGG**TCATCCTCATCCTGA**TAAACTGCAAAAGGCTGAA**GAGC-3'	15	13.4% ± 0.9%
TC06	L-98 + R-101	*CCR5*	5'-GGT**TTTGTGGGCAACATGCTGG**TCATCCTCATCCTG**ATAAACTGCAAAAGGCTGA**AGAGC-3'	14	23.6% ± 2.5%
TC07	L-100 + R-2	*CCR5*	5'-GG**TTTTGTGGGCAACATGCTG**GTCATCCTCATCCTGATA**AACTGCAAAAGGCTGAAGA**GC-3'	18	2.1% ± 1.3%
TC08	L-100 + R-99	*CCR5*	5'-GG**TTTTGTGGGCAACATGCTG**GTCATCCTCATCCTGA**TAAACTGCAAAAGGCTGAA**GAGC-3'	16	6.9% ± 0.5%
TC09	L-100 + R-101	*CCR5*	5'-GG**TTTTGTGGGCAACATGCTG**GTCATCCTCATCCTG**ATAAACTGCAAAAGGCTGA**AGAGC-3'	15	9.0% ± 1.0%
TC10	L-140 + R-2	*CCR5*	5'-GGTTT**TGTGGGCAACATGCTGGTC**ATCCTCATCCTGATA**AACTGCAAAAGGCTGAAGA**GC-3'	15	8.8% ± 2.0%
TC11	L-140 + R-99	*CCR5*	5'-GGTTT**TGTGGGCAACATGCTGGTC**ATCCTCATCCTGA**TAAACTGCAAAAGGCTGAA**GAGC-3'	13	14.4% ± 1.5%
TC12	L-140 + R-101	*CCR5*	5'-GGTTT**TGTGGGCAACATGCTGGTC**ATCCTCATCCTG**ATAAACTGCAAAAGGCTGA**AGAGC-3'	12	22.1% ± 2.4%
TC-NC	L-1 + R-2	*CCR5*	5'-GGTTT**TGTGGGCAACATGCTGGTC**ATCCTCATCCTGATA**AACTGCAAAAGGCTGAAGA**GC-3'	15	20.7% ± 0.3%
ZFNC	ZFNL + ZFNR	*CCR5*	5'-GGTTTTGTGGGCAACATGCTG**GTCATCCTCATC**CTGAT**AAACTGCAAAAG**GCTGAAGAGC-3'	5	12.8% ± 1.8%
TA01	L-92 + R-95	*AAVS1*	5'-TT**TTATCTGTCCCCTCCACCC**CACAGTGGGGCCACTAGG**GACAGGATTGGTGACAGAA**AAGC-3'	18	1.9% ± 1.4%
TA02	L-92 + R-97	*AAVS1*	5'-TT**TTATCTGTCCCCTCCACCC**CACAGTGGGGCCACTAGGGA**CAGGATTGGTGACAGAAAA**GC-3'	20	19.8% ± 0.6%
TA03	L-92 + R-136	*AAVS1*	5'-TT**TTATCTGTCCCCTCCACCC**CACAGTGGGGCCA**CTAGGGACAGGATTGGTGA**CAGAAAAGC-3'	13	30.8% ± 3.2%
TA04	L-94 + R-95	*AAVS1*	5'-TTTTA**TCTGTCCCCTCCACCCCAC**AGTGGGGCCACTAGG**GACAGGATTGGTGACAGAA**AAGC-3'	15	32.1% ± 4.6%
TA05	L-94 + R-97	*AAVS1*	5'-TTTTA**TCTGTCCCCTCCACCCCAC**AGTGGGGCCACTAGGGA**CAGGATTGGTGACAGAAAA**GC-3'	17	5.6% ± 0.8%
TA06	L-94 + R-136	*AAVS1*	5'-TTTTA**TCTGTCCCCTCCACCCCAC**AGTGGGGCCA**CTAGGGACAGGATTGGTGA**CAGAAAAGC-3'	10	22.8% ± 3.5%
TA07	L-96 + R-95	*AAVS1*	5'-TTTTATC**TGTCCCCTCCACCCCACAG**TGGGGCCACTAGG**GACAGGATTGGTGACAGAA**AAGC-3'	13	27.7% ± 4.0%
TA08	L-96 + R-97	*AAVS1*	5'-TTTTATC**TGTCCCCTCCACCCCACAG**TGGGGCCACTAGGGA**CAGGATTGGTGACAGAAAA**GC-3'	15	11.5% ± 4.3%
ZFNA	ZFNL + ZFNR	*AAVS1*	5'-TTTTATCTGTCCCCTCC**ACCCCACAGTGG**GGCCAC**TAGGGACAGGAT**TGGTGACAGAAAAGC-3'	6	21.6% ± 7.5%
TI-NC	L-3 + R-4	*IL2RG*	5'-GC**TACACGTTTCGTGTTCGGA**GCCGCTTTAACCCAC**TCTGTGGAAGTGCTCAGCA**TTGGAGTGAATGGAGCCACCCAAT-3'	15	3.3% ± 0.9%
TI01	L-3 + R-4	*IL2RG*	5'-GC**TACACGTTTCGTGTTCGGA**GCCGCTTTAACCCAC**TCTGTGGAAGTGCTCAGCA**TTGGAGTGAATGGAGCCACCCAAT-3'	15	2.6% ± 1.1%
TI02	L-3 + R-170	*IL2RG*	5'-GC**TACACGTTTCGTGTTCGGA**GCCGCTTTAACCCACTCTGTGGAAGTGCT**CAGCATTGGAGTGAATGGA**GCCACCCAAT-3'	29	0.0% ± 0.0%
TI03	L-3 + R-172	*IL2RG*	5'-GC**TACACGTTTCGTGTTCGGA**GCCGCTTTAACCCACTCTGT**GGAAGTGCTCAGCATTGGA**GTGAATGGAGCCACCCAAT-3'	20	1.5% ± 1.5%
TI04	L-3 + R-174	*IL2RG*	5'-GC**TACACGTTTCGTGTTCGGA**GCCGCTTTAACCCACTCTGTGGAAGTGCTCAGCATTGG**AGTGAATGGAGCCACCCA**AT-3'	38	0.0% ± 0.0%
TI05	L-169 + R-4	*IL2RG*	5'-GCTACACGTTTCGTGT**TCGGAGCCGCTTTAACCCA**C**TCTGTGGAAGTGCTCAGCA**TTGGAGTGAATGGAGCCACCCAAT-3'	1	0.7% ± 0.5%
TI06	L-169 + R-170	*IL2RG*	5'-GCTACACGTTTCGTGT**TCGGAGCCGCTTTAACCCA**CTCTGTGGAAGTGCT**CAGCATTGGAGTGAATGGA**GCCACCCAAT-3'	15	0.5% ± 0.5%
TI07	L-169 + R-172	*IL2RG*	5'-GCTACACGTTTCGTGT**TCGGAGCCGCTTTAACCCA**CTCTGT**GGAAGTGCTCAGCATTGGA**GTGAATGGAGCCACCCAAT-3'	6	0.7% ± 0.7%
TI08	L-169 + R-174	*IL2RG*	5'-GCTACACGTTTCGTGT**TCGGAGCCGCTTTAACCCA**CTCTGTGGAAGTGCTCAGCATTGG**AGTGAATGGAGCCACCCA**AT-3'	24	0.9% ± 0.6%
TI09	L-171 + R-4	*IL2RG*	5'-GCTACACGTT**TCGTGTTCGGAGCCGCTT**TAACCCAC**TCTGTGGAAGTGCTCAGCA**TTGGAGTGAATGGAGCCACCCAAT-3'	8	4.4% ± 4.1%
TI10	L-171 + R-170	*IL2RG*	5'-GCTACACGTT**TCGTGTTCGGAGCCGCTT**TAACCCACTCTGTGGAAGTGCT**CAGCATTGGAGTGAATGGA**GCCACCCAAT-3'	22	3.1% ± 2.2%
TI11	L-171 + R-172	*IL2RG*	5'-GCTACACGTT**TCGTGTTCGGAGCCGCTT**TAACCCACTCTGT**GGAAGTGCTCAGCATTGGA**GTGAATGGAGCCACCCAAT-3'	13	7.4% ± 1.6%
TI12	L-171 + R-174	*IL2RG*	5'-GCTACACGTT**TCGTGTTCGGAGCCGCTT**TAACCCACTCTGTGGAAGTGCTCAGCATTGG**AGTGAATGGAGCCACCCA**AT-3'	31	1.0% ± 0.9%
TI13	L-173 + R-170	*IL2RG*	5'-GCTACACGTTTCGTGTTCGGAGCCGCTT**TAACCCACTCTGTGGAAG**TGCT**CAGCATTGGAGTGAATGGA**GCCACCCAAT-3'	4	1.4% ± 1.4%
TI14	L-173 + R-174	*IL2RG*	5'-GCTACACGTTTCGTGTTCGGAGCCGCTT**TAACCCACTCTGTGGAAG**TGCTCAGCATTGG**AGTGAATGGAGCCACCCA**AT-3'	13	35.2% ± 3.3%
ZFNI	ZFNL + ZFNR	*IL2RG*	5'-GCTACACGTTTCGTGTT**CGGAGCCGCTTT**AACCC**ACTCTGTGGAAG**TGCTCAGCATTGGAGTGAATGGAGCCACCCAAT-3'	5	26.1% ± 2.2%

[1] The respective nucleases target sites are highlighted in bold and the CpG dinucleotides within the targets are underlined
[2] In shaded the optimal spacer length.
[3] Average percentage of modified alleles indicated as result of three independent experiments. SEM: standard error of the mean

Supplementary Table S2. Target sites in *CCR5* and *CCR2*

Nuclease ID	Nucleotide differences Total	Left	Right	Target sequence (5'…3')²	Spacer (bp)	Cleavage (%) at *CCR5*	Cleavage (%) at *CCR2*
***CCR5*-target locus**				5'-GGTTTTGTGGGCAACATGCTGGTCATCCTCATCCTGATAAACTGCAAAAGGCTGAAGAGC-3'			
***CCR2*-target locus¹**				5'-GGTTTTGTGGGCAACATGCTGGTC**g**TCCTCATC**tTa**ATAAACTGCAAAA**a**GCTGAAG**t**GC-3'			
TC01	2	0	2	5'-GGTTT**TGTGGGCAACATGCTGGTC**aTCCTCATCcTgATA**AACTGCAAAAgGCTGAAGa**GC-3'	15	5.7%	1.0%
TC02	1	0	1	5'-GGTTT**TGTGGGCAACATGCTGGTC**aTCCTCATCcTgA**TAAACTGCAAAAgGCTGAA**GaGC-3'	13	14.8%	13.3%
TC03	1	0	1	5'-GGTTT**TGTGGGCAACATGCTGGTC**aTCCTCATCcTg**ATAAACTGCAAAAgGCTGA**AGaGC-3'	12	21.7%	10.8%
TC04	2	0	2	5'-GGT**TTTGTGGGCAACATGCTGG**TCaTCCTCATCcTgATA**AACTGCAAAAgGCTGAAGa**GC-3'	17	4.4%	2.3%
TC05	1	0	1	5'-GGT**TTTGTGGGCAACATGCTGG**TCaTCCTCATCcTgA**TAAACTGCAAAAgGCTGAA**GaGC-3'	15	13.4%	5.1%
TC06	1	0	1	5'-GGT**TTTGTGGGCAACATGCTGG**TCaTCCTCATCcTg**ATAAACTGCAAAAgGCTGA**AGaGC-3'	14	23.6%	13.1%
TC07	2	0	2	5'-GG**TTTTGTGGGCAACATGCTG**GTCaTCCTCATCcTgATA**AACTGCAAAAgGCTGAAGa**GC-3'	18	2.1%	4.4%
TC08	1	0	1	5'-GG**TTTTGTGGGCAACATGCTG**GTCaTCCTCATCcTgA**TAAACTGCAAAAgGCTGAA**GaGC-3'	16	6.9%	2.9%
TC09	1	0	1	5'-GG**TTTTGTGGGCAACATGCTG**GTCaTCCTCATCcTg**ATAAACTGCAAAAgGCTGA**AGaGC-3'	15	9.0%	1.3%
TC10	2	0	2	5'-GGTTT**TGTGGGCAACATGCTGGTC**aTCCTCATCcTgATA**AACTGCAAAAgGCTGAAGa**GC-3'	15	8.8%	2.1%
TC11	1	0	1	5'-GGTTT**TGTGGGCAACATGCTGGTC**aTCCTCATCcTgA**TAAACTGCAAAAgGCTGAA**GaGC-3'	13	14.4%	15.4%
TC12	1	0	1	5'-GGTTT**TGTGGGCAACATGCTGGTC**aTCCTCATCcTg**ATAAACTGCAAAAgGCTGA**AGaGC-3'	12	22.1%	15.1%
TC-NC	2	0	2	5'-GGTTT**TGTGGGCAACATGCTGGTC**aTCCTCATCcTgATA**AACTGCAAAAgGCTGAAGa**GC-3'	15	20.7%	3.3%
ZFNC	2	1	1	5'-GGTTTTGTGGGCAACATGCTG**GTCaTCCTCATC**cTgAT**AAACTGCAAAAg**GCTGAAGaGC-3'	5	12.8%	18.3%

¹ Differences to *CCR5* are highlighted in lower case bold letters.

² The *CCR5*-specific nuclease target sites are highlighted in bold. Letters in lower case represent nucleotides not conserved in *CCR2*.

Supplementary Table S3. Off-target sites of *CCR5* - and *AAVS1* -specific zinc finger nucleases predicted by PROGNOS tool

Nuclease ID	PROGNOS prediction rankings		Nucleotide differences			Closest Gene¹	chromosome	Match Type	Left target (5'-->3')²	Spacer (bp)	Right target (5'-->3')²	NHEJ detected	References
	Homology	Conserved Gs	Total	Left	Right								
	1	1	0	0	0	*CCR5*	chr3	L-5-R	GTCATCCTCATC	5	AAACTGCAAAAG	Yes	26, 27, 51
	2	5	2	1	1	*CCR2*	chr3	L-5-R	GTCgTCCTCATC	5	AAACTGCAAAAa	Yes	26, 27,51
	3	45	3	2	1	*BTBD10*	chr11	L-5-R	GTttTCCTCATC	5	AAACTGCAAAAt	Yes	27, 51
	4	16	3	2	1	*SYT10*	chr12	L-6-R	GTCATCCcaATC	6	AAACTGaAAAAG	No	27
	5	2	3	2	1	*GALNT13*	chr2	R-5-L	CTTcTGCtGTTT	5	GcTGAGGATGAC	No	27
	6	3	3	1	2	*DGKK*	chrX	L-5-R	cTCATCCTCATC	5	AcAaTGCAAAAG	No	27
	7	46	3	2	1	*CEP112*	chr17	R-5-L	gTTTTGCAGTTc	5	GATGAaGATGAC	Yes	27
	8	4	3	1	2	*PSAT1*	chr9	R-6-L	CTTTTGCAGTcT	6	GtTGAGGtTGAC	Yes	27
	9	26	4	3	1	*CWF19L2*	chr11	R-5-L	CcTTTtCAGcTT	5	GATGAtGATGAC	N/A	N/A
	10	6	4	3	1	*LPCAT2*	chr16	R-6-L	CTTcaGCtGTTT	6	GATGAGGATGgC	No	51
	11	195	4	3	1	*ZNF587*	chr19	L-6-R	GTaATCaTCAgC	6	AcACTGCAAAAG	No	51
	12	196	4	1	3	*ZNF417*	chr19	R-6-L	CTTTTGCAGTgT	6	GcTGAtGATtAC	No	51
	13	27	4	1	3	*SPTB*	chr14	L-6-R	GTCATCCgCATC	6	gAACTGgAAAAa	No	27
	14	417	4	1	3	*LOC100507217*	chr15	R-6-L	tTTTTGCAGTTT	6	tATGcGtATGAC	No	51
	15	28	4	3	1	*TMEM114*	chr16	R-5-L	CTTcTaCtGTTT	5	GATGAtGATGAC	N/A	N/A
	16	7	4	1	3	*TMOD1*	chr9	L-5-R	GTCATCCTCcTC	5	AAtCTGaAgAAG	No	51
ZFNC	17	197	4	1	3	*TACR3*	chr4	L-5-R	GTCATCtTCATC	5	AAACTGtAAAgt	Yes	27, 51
	18	8	4	1	3	*ACOXL*	chr2	R-5-L	CTTTTGCAGTgT	5	GATGgGGtaGAC	No	51
	19	9	4	3	1	*MTM1*	chrX	R-6-L	CTgTTGCtGTgT	6	GATGAGGATGAt	No	51
	20	198	4	1	3	*PCSK2*	chr20	R-5-L	CTTcTGCAGTTT	5	GATGAcatTGAC	N/A	N/A
	21	29	4	1	3	*PIWIL2*	chr8	L-5-R	GTCATCCTCATa	5	AAACTGCcttAG	No	27
	22	10	4	1	3	*IFLTD1*	chr12	L-6-R	cTCATCCTCATC	6	AAgggGCAAAAG	No	51
	23	30	4	3	1	*NPAS3*	chr14	R-5-L	CTTTgGCAGaTa	5	GATGAGcATGAC	N/A	N/A
	24	31	4	3	1	*MAP4*	chr3	L-6-R	GcCATCCTCAgg	6	AAACTGCAAcAG	No	N/A
	25	32	4	1	3	*SLC4A8*	chr12	R-6-L	CTTTTGCAtTTT	6	GATGAGGATtta	No	27
	26	199	4	3	1	*DAB1*	chr1	L-5-R	aTCATCCTCAgt	5	AAACTGCAAAAt	N/A	N/A
	27	200	4	1	3	*PTPRG*	chr3	R-6-L	CaTTTGCAGTTT	6	GATGAtGcTtAC	N/A	N/A
	28	11	4	1	3	*WDPCP*	chr2	L-6-R	GTCATCCTCAgC	6	AcACaGgAAAAG	No	51
	29	33	4	3	1	*KCNB2*	chr8	L-5-R	aTgtTCCTCATC	5	AAACTGCAAAtG	Yes	27, 51
	30	34	4	1	3	*SYNDIG1L*	chr14	L-6-R	GTCATCaTCATC	6	ttACaGCAAAAG	N/A	N/A
	31	12	4	3	1	*WWOX*	chr16	R-6-L	CTTTaGCAaTTg	6	GAgGAGGATGAC	No	27
	32	201	4	3	1	*MIR100HG*	chr11	L-5-R	cTgAgCCTCATC	5	AAACTGCAAAAc	N/A	N/A
	33	13	4	3	1	*CSNK1G3*	chr5	L-5-R	GcCtTCCcCATC	5	AAAgTGCAAAAG	Yes	51

Nuclease ID	PROGNOS prediction rankings		Nucleotide differences			Closest Gene¹	chromosome	Match Type	Left target (5'-->3')²	Spacer (bp)	Right target (5'-->3')²	NHEJ detected	References
	Homology	Conserved Gs	Total	Left	Right								
	1	1	0	0	0	*AAVS1*	chr19	R-6-L	ACCCCACAGTGG	6	TAGGGACAGGAT	Yes	21
	N/A	N/A	6	4	2	*CHRAC1*	chr8	R-6-L	cTCCTGgCCCag	6	CCACTGTGGGtg	Yes	21
	1641	662	6	3	3	*ANXA8L2*	chr10	L-6-R	caCCCACAGgGG	6	cAGGGcCAGGAc	No	21
	19	31	4	3	1	*RGS12*	chr4	R-5-L	tTCCTGTCCtTt	5	CCACTGTGGGtT	N/A	21
	N/A	N/A	5	5	0	*ATRNL1*	chr10	L-5-R	caCCCACAGatt	5	TAGGGACAGGAT	Yes	21
	29	5	4	2	2	*BEGAIN*	chr14	R-6-L	gTCCTGTCCCTg	6	CCACaGTGGGGg	Yes	21
	2	2	2	1	1	*LINC00548*	chr13	L-6-R	ACCCCACAGTaG	6	TAGGGACAGGAa	Yes	N/A
	3	4	3	1	2	*LOC100130539*	chr10	L-5-R	ACgCCACAGTGG	5	cAGGGACAGGtT	No	N/A
	4	10	4	3	1	*LHPP*	chr10	L-5-R	tCCCaACAGcGG	5	TAGGGcCAGGAT	No	N/A
	5	11	4	3	1	*SAG*	chr2	L-6-R	ACCCCACctTGt	6	TAGGcACAGGAT	No	N/A
	7	25	4	3	1	*FOXP1*	chr3	L-5-R	gCCtCcCAGTGG	5	TAGaGACAGGAT	No	N/A
ZFNA	16	3	4	1	3	*H19*	chr11	L-5-R	tCCCCACAGTGG	5	gAGGGcCAGGAg	Yes	N/A
	30	6	4	2	2	*SYNPO*	chr5	L-5-R	tCCCCACAaTGG	5	TtGGGcCAGGAT	No	N/A
	32	7	4	2	2	*MEGF6*	chr1	L-5-R	ACCCCgCAGaGG	5	gAGGGACAGGgT	No	N/A
	36	8	4	2	2	*TK2*	chr16	L-5-R	tCCCCACAcTGG	5	TAGGGgCAGGAg	No	N/A
	42	9	4	2	2	*ZNF521*	chr18	L-6-R	ACCCCACAGatG	6	TgGGGcCAGGAT	No	N/A
	8	26	4	3	1	*CCDC33*	chr15	R-6-L	AcCCTGTCtCTg	6	CCACTGTGGGaT	No	N/A
	9	27	4	3	1	*GAS2L3*	chr12	R-6-L	AaaCTGTCCgTA	6	CCAtTGTGGGGT	No	N/A
	10	28	4	3	1	*MIR4299*	chr11	R-6-L	AaCCTGctCCTA	6	CCACTGTGGGcT	No	N/A
	11	12	4	3	1	*SLC6A19*	chr5	R-5-L	AgCCTGTgCCTc	5	CCcCTGTGGGGT	N/A	N/A
	13	14	4	1	3	*RAB11FIP2*	chr10	L-5-R	ACCCCtCAGTGG	5	TgGGGtCAGaAT	No	N/A
	14	15	4	1	3	*NMT2*	chr10	R-5-L	ATCCTGTCCCTc	5	CCcCTGTGaGGc	No	N/A
	17	16	4	1	3	*CLU*	chr8	L-5-R	ACCCCACAGTGa	5	TAGGtcCtGGAT	No	N/A
	21	17	4	1	3	*FRS3*	chr6	L-5-R	ACCCCACAGTGa	5	aAGaGAaAGGAT	No	N/A

¹ Lines highlighted in grey represent experimentally validated off-target sites (references indicated).
² Differences to *CCR5* or *AAVS1* are highlighted in lower case bold letters.

Supplementary Table S4. Off-target sites of *CCR5*- and *AAVS1*-specific TALENs predicted by PROGNOS tool

Nuclease ID	Ranking[1]	Nucleotide differences Total	Left	Right	Closest Gene	chromosome	Match Type	Left target (5'-->3')[2]	Spacer (bp)	Right target (5'-->3')[2]
	1	0	0	0	*CCR5*	chr3	L-15-R	TGTGGGCAACATGCTGGTC	15	AACTGCAAAAGGCTGAAGA
	2	7	2	5	*AUTS2*	chr7	L-27-R	TGaGGGCcACATGCTGGTC	27	AAgTGgAcAcGGgTGAAGA
	3	9	4	5	*CDH8*	chr16	R-11-L	TCccCAcCCTTTTGgAGTT	11	GACCAGCATtTTGgttAaA
	4	10	5	5	*CAPZA3*	chr12	L-15-L	TGgGGGCAcaATaCTGtTC	15	GACttGCATGTTagaCACA
	5	9	4	5	*NRXN1*	chr2	L-16-L	TaTGGtCAgCATGCTGGaC	16	GcCCAGCAgGcTGCgCtCA
	6	10	5	5	*HMGCLL1*	chr6	L-12-L	TGTGacCAcCcTcCTGGTC	12	aACaAtCATGTTcCCaACA
	7	10	5	5	*PADI3*	chr1	R-28-L	TCcTCAcCCTgTaaCAGTT	28	GAgCtGCATGTgcCCCAtA
TC-NC	8	9	5	4	*AOAH*	chr7	L-25-R	TGTaGaCAACATGCatGcC	25	AtCTGCtAAAGGCTaAAaA
	9	9	5	4	*NRN1*	chr6	R-30-L	TCTcCAGgCTTTTcaAaTT	30	GACCAGCATtTTcCtCcCA
	10	10	5	5	*PRPS2*	chrX	L-30-L	TGTGGcCAAtATGCTGtcg	30	GACCtGCATGcTtCtCAgA
	11	10	5	5	*LRRC8D*	chr1	L-13-R	TcTGGGCAACATagTGaTa	13	AAtTtaAAAAGcCTGAAaA
	12	9	5	4	*LIPC*	chr15	L-20-L	TtTGttCAtCtTGCTGGTC	20	GACCgGCgTGTgGCCCcCA
	13	10	5	5	*PDE9A*	chr21	L-19-L	TGTGGtCtcCATGCTGcTg	19	GttCAGgAacTTGCCCACA
	14	9	4	5	*CCR8*	chr3	L-15-R	TcTGGGaAACAgcCTGGTC	15	gtCTGCAAgAaGCTGAgGA
	15	9	4	5	*C11orf82*	chr11	R-15-R	TaTTCAGCaaaTTGCAGTT	15	cACTGtActAGGtTGAAGA
	N/A[3]	2	0	2	*CCR2*	chr3	L-15-R	TGTGGGCAACATGCTGGTC	15	AACTGCAAAAaGCTGAAGt
	1	0	0	0	*CCR5*	chr3	L-14-R	TTTGTGGGCAACATGCTGG	14	ATAAACTGCAAAAGGCTGA
	2	1	0	1	*CCR2*	chr3	L-14-R	TTTGTGGGCAACATGCTGG	14	ATAAACTGCAAAAaGCTGA
	3	9	5	4	*AOAH*	chr7	L-24-R	cTTGTaGaCAACATGCatG	24	tTAAtCTGCtAAAGGCTaA
	4	8	5	3	*SFMBT2*	chr10	L-11-L	TTTGgGGatAACATGaTGa	11	CCAtCATGTatCCCACAAA
	5	10	5	5	*FAM84A*	chr2	L-21-L	TTgaTGGGCAACcaaCTGG	21	aCAGCgaGTTGCCCACAgg
	6	10	5	5	*TBL1X*	chrX	L-18-L	TaaGTaGGCAACATcCTGt	18	CtAtCtTcTTGCCCAaAAA
	7	10	5	5	*LOC100422737*	chr6	L-15-L	TTTGTaGGCgACATGaTcc	15	CCAtagTGTTGgCCAaAAA
TC06	8	10	5	5	*CAPZA3*	chr12	L-19-L	TTTGgGGGCAcaATaCTGt	19	CttGCATGTTagaCACAAA
	9	9	5	4	*LOC728084*	chr12	L-17-R	TcTGaGGGCAACATcCTac	17	ATAgACTGCAcAAGagTGA
	10	8	3	5	*LOC283038*	chr10	L-17-L	TTTGaGGGgAAgATGCTGG	17	gCAaCATGTTGgaCACAcA
	11	10	5	5	*CHADL*	chr22	L-15-L	TTTcTGGGCAcCAaGCaGt	15	CCAGggTGTTtCCCAgAcA
	12	8	5	3	*TRMT44*	chr4	L-22-L	TTTGaGGGggAaATGCTtG	22	CCAGCgTGgTGCCCACAcA
	13	9	4	5	*HS3ST5*	chr6	R-18-L	TCtcCCTcTTGCAGgTTAT	18	CCAGCATcgTGattACAAA
	14	10	5	5	*NLGN1*	chr3	L-22-L	TTTcaaGGCttCATGCTGG	22	CCAtCATGTattCCACAgA
	15	8	4	4	*LOC100133091*	chr7	R-25-L	TCAGCtTTcatCAGTTTAT	25	CCccCATGTTGCCCACgAg
	16	10	5	5	*RALYL*	chr8	L-19-L	TTTGcaGGtAACATGaTtG	19	CCAtaATcTTGgCCAaAAA

Nuclease ID	Ranking[1]	Nucleotide differences Total	Left	Right	Closest Gene	chromosome	Match Type	Left target (5'-->3')[2]	Spacer (bp)	Right target (5'-->3')[2]
	1	0	0	0	*AAVS1*	chr19	L-15-R	TCTGTCCCCTCCACCCCAC	15	GACAGGATTGGTGACAGAA
	2	7	4	3	*SHANK3*	chr22	L-18-R	TCTGctCCCcCCACCCCcC	18	GACtGGAgTGGgGACAGAA
	3	8	5	3	*MRM1*	chr17	L-24-L	TCTGcCCCCaaCACtCCAg	24	GgGGGGTGGAGtGGAgAGA
	4	8	5	3	*LOC283867*	chr16	L-21-L	TgTGTCCCCTCCcCttgAC	21	GTGGGGTccAGGGGACAtA
	5	8	4	4	*SERPINE1*	chr7	L-24-R	TCaGcCaCCaCCACCCCAC	24	GACAaGgTTGtTGACAcAA
	6	8	4	4	*LOC100144595*	chr2	L-26-R	TCTtTCCaCTCCACCtCtC	26	GACAGGATTGGTGAgtagA
	7	8	4	4	*RAB4B-EGLN2*	chr19	L-10-L	TCTaTCCCCcCCAaCCCcC	10	aTGGGcTaGAGGGGgCAGA
	8	9	5	4	*CDKL5*	chrX	L-13-R	cCTcTCCCCTCtACCtCcC	13	GttAGGcTTGGTGAgAGAA
	9	10	5	5	*HECW1*	chr7	L-27-R	cCTcTCCCCTCCcCtCCcC	27	GACAGGgTTtGTGtCAGtg
	10	10	5	5	*CPN1*	chr10	L-11-R	cCacTCCCtcCCACCCCAC	11	aAtAGGATTGGgGgCAGgA
	11	10	5	5	*KIAA0556*	chr16	R-21-L	TTCaGTgACCAcTCCTaTa	21	GTGGGGgtGAGaGGAggGA
TA04	12	10	5	5	*TMEM51*	chr1	L-24-L	TgTcTaCCCTtCACCCaAC	24	GgGGGGgGGAGGGGggAGg
	13	10	5	5	*ORMDL3*	chr17	R-13-R	TTCTcTttCCAATaCTGTg	13	GACAGGggTGGTGgCtGgA
	14	10	5	5	*HSP90AB2P*	chr4	R-17-L	cTCTaTCtCCAAaCCTGTa	17	GTGGGGaGGAtGttACAaA
	15	9	4	5	*CACUL1*	chr10	R-14-R	TcCTtTCcCCAATgCTGTC	14	GACAaGATTGtTcACcaAA
	16	9	4	5	*WDR3*	chr1	R-24-L	TTCTGTagCgAATCCTtTC	24	GcGGGaaGGAaGaGACAGA
	17	9	5	4	*ZBTB9*	chr6	L-23-L	cCTtTCCCaTCCcaCCCAC	23	GaGataTGGAGGGGACAGA
	18	10	5	5	*NFX1*	chr9	R-20-R	cTtTGTCcCtAAcCCTGTC	20	cACAGagTaGGTGAaAGAA
	19	9	4	5	*KCNK3*	chr2	L-20-R	TtTGTCCaCaCCAtCCCAC	20	GACcaGAaTGGgGACAGAg
	20	9	5	4	*DUSP10*	chr1	L-11-R	TCTcTtCCCTCCtCCCagC	11	GACAtGAgTGGTGACttAA
	21	10	5	5	*HACE1*	chr6	R-20-R	TTtTGTaAtaAATCCaGTC	20	GACtaGATTGaTGggAGAA
	22	9	4	5	*XYLT1*	chr16	L-12-L	TCTGcCCaCaCCcCCCCAC	12	GgGttGTGcAGGGGACAcA
	23	9	5	4	*LRSAM1*	chr9	R-11-L	TTCaGcCACCtcTgCTGTC	11	GctGGGTaGAGGtGACAGA

[1] Potential off-target were chosen to contain a mixture of the top-ranked sites from the RVD-5TC and Homology-5TC PROGNOS algorithms.

[2] Differences to *CCR5* or *AAVS1* are highlighted in lower case bold letters.

[3] The *CCR2* off-target site lacks the 5'-T in the right sub-site, which automatically excludes the site when applying the PROGNOS algorythm RVD-5TC.

Supplementary Table S5. Results of the SMRT sequencing

Nuclease ID	Nucleotide differences T[1]	L	R	Closest Gene	Match Type	hg19 Coordinates	Empty Vector indels	total	mutation frequency	indels	total	mutation frequency	*p*-value[2]
**Experiment #1 - *CCR5***													
ZFNC	0	0	0	*CCR5*	L-5-R	chr3:46414544	0	770		**111**	**398**	**27.89%**	**1.00E-33**
	2	1	1	*CCR2*	L-5-R	chr3:46399205	0	7855		**365**	**3695**	**9.88%**	**7.00E-89**
TC-NC	0	0	0	*CCR5*	L-15-R	chr3:46414528	0	770		**80**	**700**	**11.43%**	**4.00E-21**
	7	2	5	*AUTS2*	L-27-R	chr7:69824484	0	417		0	728		
	9	4	5	*CDH8*	R-11-L	chr16:62697815	0	480		0	910		
	10	5	5	*CAPZA3*	L-15-L	chr12:19069027	0	235		0	355		
	9	4	5	*NRXN1*	L-16-L	chr2:49998175	0	1264		1	5091	0.02%	1.60E-01
	10	5	5	*HMGCLL1*	L-12-L	chr6:55393918	0	733		0	1260		
	10	5	5	*PADI3*	R-28-L	chr1:17587769	0	1067		0	1635		
	9	5	4	*AOAH*	L-25-R	chr7:36783620	0	1190		0	1846		
	9	5	4	*NRN1*	R-30-L	chr6:5894870	0	635		0	960		
	10	5	5	*PRPS2*	L-30-L	chrX:12827379	0	1122		0	1912		
	10	5	5	*LRRC8D*	L-13-R	chr1:90419966	0	708		0	1403		
	9	5	4	*LIPC*	L-20-L	chr15:58836266	0	1017		0	1919		
	10	5	5	*PDE9A*	L-19-L	chr21:44089889	0	1235		0	1860		
	9	4	5	*CCR8*	L-15-R	chr3:39373972	0	987		0	1851		
	9	4	5	*C11orf82*	R-15-R	chr11:82667987	0	1195		2	4334	0.05%	7.86E-02
	2	0	2	*CCR2*	L-15-R	chr3:46399189	0	7855		**5**	**6761**	**0.07%**	**1.30E-02**
TC06	0	0	0	*CCR5*	L-14-R	chr3:46414526	0	770		**175**	**813**	**21.53%**	**1.00E-47**
	2	0	2	*CCR2*	L-14-R	chr3:46399187	0	7855		**238**	**7737**	**3.08%**	**3.00E-55**
	9	5	4	*AOAH*	L-24-R	chr7:36783618	0	1190		0	2848		
	8	5	3	*SFMBT2*	L-11-L	chr10:7305526	0	554		0	2731		
	10	5	5	*FAM84A*	L-21-L	chr2:14774101	0	1413		0	1680		
	10	5	5	*TBL1X*	L-18-L	chrX:9227692	0	803		**3**	**2551**	**0.12%**	**4.16E-02**
	10	5	5	*LOC100422737*	L-15-L	chr6:107261533	0	821		0	934		
	10	5	5	*CAPZA3*	L-19-L	chr12:19069025	0	235		0	322		
	9	5	4	*LOC728084*	L-17-R	chr12:89421523	0	1686		0	1762		
	8	3	5	*LOC283038*	L-17-L	chr10:127381295	0	1142		0	1282		
	10	5	5	*CHADL*	L-15-L	chr22:41625772	0	824		0	955		
	8	5	3	*TRMT44*	L-22-L	chr4:8528150	0	1417		**4**	**3473**	**0.12%**	**2.27E-02**
	9	4	5	*HS3ST5*	R-18-L	chr6:114639789	1	2077	0.00048	0	2411		
	10	5	5	*NLGN1*	L-22-L	chr3:173623195	0	1601		0	1922		
	8	4	4	*LOC100133091*	R-25-L	chr7:76237984	0	1691		0	1705		
	10	5	5	*RALYL*	L-19-L	chr8:84679140	0	699		0	664		

Nuclease ID	Nucleotide differences T[1]	L	R	Closest Gene	Match Type	hg19 Coordinates	Empty Vector indels	total	mutation frequency	indels	total	mutation frequency	*p*-value[2]
**Experiment #2 - *CCR5***													
ZFNC	0	0	0	*CCR5*	L-5-R	chr3:46414544	0	770		**93**	**646**	**14.40%**	**7.00E-25**
	2	1	1	*CCR2*	L-5-R	chr3:46399205	0	7855		**70**	**881**	**7.95%**	**2.00E-18**
	3	2	1	*BTBD10*	L-5-R	chr11:13485162	0	313		**66**	**1043**	**6.33%**	**6.00E-17**
	4	1	3	*TACR3*	L-5-R	chr4:104555726	0	720		**231**	**1827**	**12.64%**	**6.00E-58**
	4	2	2	*KRR1*	R-5-L	chr12:75963450	PCR Failure			PCR Failure			
	4	3	1	*KCNB2*	L-5-R	chr8:73736816	0	802		**18**	**1638**	**1.10%**	**1.00E-05**
	7	2	5	*KDM2A*	R-5-L	chr11:66963780	0	970		**231**	**2694**	**8.58%**	**2.00E-55**
	4	2	2	*WBSCR17*	R-6-L	chr7:70919318	0	739		**41**	**1518**	**2.70%**	**5.00E-11**
TC-NC	0	0	0	*CCR5*	L-15-R	chr3:46414528	0	770		**23**	**293**	**7.85%**	**3.00E-07**
	7	2	5	*AUTS2*	L-27-R	chr7:69824484	0	417		0	348		
	9	4	5	*CDH8*	R-11-L	chr16:62697815	0	480		0	437		
	10	5	5	*CAPZA3*	L-15-L	chr12:19069027	0	235		0	297		
	9	4	5	*NRXN1*	L-16-L	chr2:49998175	0	1264		0	1141		
	10	5	5	*HMGCLL1*	L-12-L	chr6:55393918	0	733		0	645		
	10	5	5	*PADI3*	R-28-L	chr1:17587769	0	1067		0	1231		
	9	5	4	*AOAH*	L-25-R	chr7:36783620	0	1190		0	1054		
	9	5	4	*NRN1*	R-30-L	chr6:5894870	0	635		0	921		
	10	5	5	*PRPS2*	L-30-L	chrX:12827379	0	1122		0	973		
	10	5	5	*LRRC8D*	L-13-R	chr1:90419966	0	708		0	1214		
	9	5	4	*LIPC*	L-20-L	chr15:58836266	0	1017		0	1461		
	10	5	5	*PDE9A*	L-19-L	chr21:44089889	0	1235		0	1565		
	9	4	5	*CCR8*	L-15-R	chr3:39373972	0	987		0	1191		
	9	4	5	*C11orf82*	R-15-R	chr11:82667987	0	1195		**5**	**4309**	**0.12%**	**1.27E-02**
	2	0	2	*CCR2*	L-15-R	chr3:46399189	0	7855		**5**	**6875**	**0.07%**	**1.30E-02**

Nuclease ID	Nucleotide differences T[1]	L	R	Closest Gene	Match Type	hg19 Coordinates	Empty Vector indels	total	mutation frequency	indels	total	mutation frequency	*p*-value[2]
**Experiment #1 - *AAVS1***													
	0	0	0	*AAVS1*	R-6-L	chr19:55627120	0	985		**88**	**968**	**9.10%**	**1.30E-22**
	6	4	2	*CHRAC1*	R-6-L	chr8:141507034	0	633		**20**	**677**	**3.00%**	**3.10E-06**
	6	3	3	*ANXA8L2*	L-6-R	chr10:47635367	1	1484	0.07%	0	1615		
	4	3	1	*RGS12*	R-5-L	chr4:3303432	PCR Failure				PCR Failure		
	5	5	0	*ATRNL1*	L-5-R	chr10:117758695	0	1599		**46**	**1241**	**3.70%**	**2.90E-12**
	4	2	2	*BEGAIN*	R-6-L	chr14:101033118	0	864		**32**	**727**	**4.40%**	**4.30E-09**
	2	1	1	*LINC00548*	L-6-R	chr13:40779943	0	611		**27**	**650**	**4.20%**	**6.60E-08**
	3	1	2	*LOC100130539*	L-5-R	chr10:44698021	0	1438		0	1230		
	4	3	1	*LHPP*	L-5-R	chr10:126241979	0	40		0	42		
	4	3	1	*SAG*	L-6-R	chr2:234242672	0	1537		0	1193		
	4	3	1	*FOXP1*	L-5-R	chr3:71561355	0	349		0	378		
ZFNA	4	1	3	*H19*	L-5-R	chr11:2047066	0	601		**5**	**390**	**1.30%**	**1.20E-02**
	4	2	2	*SYNPO*	L-5-R	chr5:149981873	0	614		0	848		
	4	2	2	*MEGF6*	L-5-R	chr1:3440064	0	744		0	762		
	4	2	2	*TK2*	L-5-R	chr16:66569551	0	705		0	763		
	4	2	2	*ZNF521*	L-6-R	chr18:22999787	0	780		0	929		
	4	3	1	*CCDC33*	R-6-L	chr15:74546976	0	89		0	279		
	4	3	1	*GAS2L3*	R-6-L	chr12:101079155	0	47		0	63		
	4	3	1	*MIR4299*	R-6-L	chr11:11689693	0	1163		0	1749		
	4	3	1	*SLC6A19*	R-5-L	chr5:1180771	PCR Failure				PCR Failure		
	4	1	3	*RAB11FIP2*	L-5-R	chr10:119572401	0	652		0	834		
	4	1	3	*NMT2*	R-5-L	chr10:15227469	0	1101		0	1338		
	4	1	3	*CLU*	L-5-R	chr8:27446702	1	378	0.26%	0	398		
	4	1	3	*FRS3*	L-5-R	chr6:41727782	0	901		0	1016		

Nuclease ID	Nucleotide differences T[1]	L	R	Closest Gene	Match Type	hg19 Coordinates	Empty Vector indels	total	mutation frequency	indels	total	mutation frequency	*p*-value[2]
	0	0	0	*AAVS1*	R-15-L	chr19:55627109	0	1084		**153**	**1217**	**13.00%**	**7.40E-39**
	7	4	3	*SHANK3*	L-18-R	chr22:51139499	1	717	0.14%	1	461	0.22%	3.80E-01
	8	5	3	*MRM1*	L-24-L	chr17:35084432	3	523	0.57%	1	827	0.12%	
	8	5	3	*LOC283867*	L-21-L	chr16:65705836	0	1215		0	1454		
	8	4	4	*SERPINE1*	L-24-R	chr7:100769608	0	983		0	35		
	8	4	4	*LOC100144595*	L-26-R	chr2:155321871	0	780		0	745		
	8	4	4	*RAB4B-EGLN2*	L-10-L	chr19:41319122	0	224		0	454		
	9	5	4	*CDKL5*	L-13-R	chrX:18438490	0	1213		0	1426		
	10	5	5	*HECW1*	L-27-R	chr7:43424877		PCR Failure			PCR Failure		
	10	5	5	*CPN1*	L-11-R	chr10:101836505	0	741		**29**	**22513**	**0.13%**	**3.60E-08**
	10	5	5	*KIAA0556*	R-21-L	chr16:27592811	0	212		0	347		
TA04	10	5	5	*TMEM51*	L-24-L	chr1:15490282	0	201		0	261		
	10	5	5	*ORMDL3*	R-13-R	chr17:38076475	0	552		0	803		
	10	5	5	*HSP90AB2P*	R-17-L	chr4:12840351	0	1078		0	1809		
	9	4	5	*CACUL1*	R-14-R	chr10:120434301	0	844		0	1793		
	9	4	5	*WDR3*	R-24-L	chr1:118483739	0	1070		0	1473		
	9	5	4	*ZBTB9*	L-23-L	chr6:33425193	0	1188		0	1982		
	10	5	5	*NFX1*	R-20-R	chr9:33366385	0	1055		0	1211		
	9	4	5	*KCNK3*	L-20-R	chr2:26928594	0	452		0	726		
	9	5	4	*DUSP10*	L-11-R	chr1:221693332	0	978		0	1544		
	10	5	5	*HACE1*	R-20-R	chr6:105034857	0	65		0	80		
	9	4	5	*XYLT1*	L-12-L	chr16:17287360	0	703		0	1158		
	9	5	4	*LRSAM1*	R-11-L	chr9:130229886	0	759		0	1191		

[1] T, total; L, left target sub-site; R, right target sub-site.
[2] Statistically significant indels events are highlighted in bold.

Supplementary Table S6. Mutations induced by *CCR5*-specific designer nucleases

Nuclease ID	Sequence Reads[1]	Mutation type	Frequency (n° reads)
ZFNC	TTCATCTTTGGTTTTGTGGGCAACATGCTGGGTCATCCTCATCCTGATAAACTGCAAAAGGCTGAAGAGCATGACTGACATCTACCTGC	WT	
	TTCATCTTTGGTTTTGTGGGCAACATGCTGGTCATCCTCATC**ctgat**CTGATAAACTGCAAAAGGCTGAAGAGCATGACTGACATCTA	+5	x56
	TTCATCTTTGGTTTTGTGGGCAACATGCTGGTCATCCTCATC**tgat**CTGATAAACTGCAAAAGGCTGAAGAGCATGACTGACATCTAC	+4	x6
	TTCATCTTTGGTTTTGTGGGCAACATGCTGGTCATCCTCATCC**tgat**TGATAAACTGCAAAAGGCTGAAGAGCATGACTGACATCTAC	+4	x5
	TTCATCTTTGGTTTTGTGGGCAACATGCTGGTCATCCTCATCC**tga**TGATAAACTGCAAAAGGCTGAAGAGCATGACTGACATCTACC	+3	x9
	TTCATCTTTGGTTTTGTGGGCAACATGCTGGTCATCCTCATC**ctga**CTGATAAACTGCAAAAGGCTGAAGAGCATGACTGACATCTAC	+4	x3
	TTCATCTTTGGTTTTGTGGGCAACATGCTGG------------------------/ /-------------------------TCA	Δ204	x2
	TTCATCTTTGGTTTTGTGGGCAACATGCTGGTCATCCTCATCCTGAT**ctgat**AACTGCAAAAGGCTGAAGAGCATGACTGACATCTAC	+4 (Δ1+5)	x6
	TTCATCTTTGGTTTTGTGGGCAACATGCTGGTCATCCTCATCCTGAT**gat**AACTGCAAAAGGCTGAAGAGCATGACTGACATCTACCT	+2 (Δ1+3)	x6
	TTCATCTTTGGTTTTGTGGGCAACATGCTGGTCATCCTC------ATAAACTGCAAAAGGCTGAAGAGCATGACTGACATCTACCTGC	Δ6	x2
TC-NC	GTGTTCATCTTTGGTTTTGTGGGCAACATGCTGGTCATCCTCATCCTGATAAACTGCAAAAGGCTGAAGAGCATGACTGACATCTACC	WT	
	GTGTTCATCTTTGGTTTTGTGGGCAACATGCTGG------TCATCCTGATAAACTGCAAAAGGCTGAAGAGCATGACTGACATCTACC	Δ6	x13
	GTGTTCATCTTTGGTTTTGTGGGCAACATGCTGGTCATCC**tca**TCATCCTGATAAACTGCAAAAGGCTGAAGAGCATGACTGACATCT	+3	x4
	GTGTTCATCTTTGGTTTTGTGGGCAACATGCTGGTCATCC---TCCTGATAAACTGCAAAAGGCTGAAGAGCATGACTGACATCTACC	Δ3	x2
	GTGTTCATCTTTGGTTTTGTGGGCAACAT-------------------------/ /--------------------------GCT	Δ238	x2
TC06	GTGTTCATCTTTGGTTTTGTGGGCAACATGCTGGTCATCCTCATCCTGATAAACTGCAAAAGGCTGAAGAGCATGACTGACATCTACC	WT	
	GTGTTCATCTTTGGTTTTGTGGGCAACATGCTGG------TCATCCTGATAAACTGCAAAAGGCTGAAGAGCATGACTGACATCTACC	Δ6	x16
	GTGTTCATCTTTGGTTTTGTGGGCAACATGCTGGTCATC**ctca**CTCATCCTGATAAACTGCAAAAGGCTGAAGAGCATGACTGACATC	+4	x5
	GTGTTCATCTTTGGTTTTGTGGGCAACATGCTGGTCATCCTC------ATAAACTGCAAAAGGCTGAAGAGCATGACTGACATCTACC	Δ6	x4
	GTGTTCATCTTTGGTTTTGTGGGCAACATGCTGGTCATCCtcaTCATCCTGATAAACTGCAAAAGGCTGAAGAGCATGACTGACATCT	+3	x4
	GTGTTCATCTTTGGTTTTGTGGGCAACATGCTGGTCA---TCATCCTGATAAACTGCAAAAGGCTGAAGAGCATGACTGACATCTACC	Δ3	x4
	GTGTTCATCTTTGGTTTTGTGGGCAACATGCTGG---------TCCTGATAAACTGCAAAAGGCTGAAGAGCATGACTGACATCTACC	Δ9	x4
	GTGTTCATCTTTGGTTTTGTGGGCAACATGCTGGTCATCCTC--------AAACTGCAAAAGGCTGAAGAGCATGACTGACATCTACC	Δ8	x3
	GTGTTCATCTTTGGTTTTGTGGGCAACATGCTGGTCAT-------CTGATAAACTGCAAAAGGCTGAAGAGCATGACTGACATCTACC	Δ7	x3
	GTGTTCATCTTTGGTTTTGTGGGCAACAT-------------------------/ /--------------------------GCT	Δ238	x3
	GTGTTCATCTTTGGTTTTGTGGGCAACATGCTGGTCAT-----TCCTGATAAACTGCAAAAGGCTGAAGAGCATGACTGACATCTACC	Δ5	x2
	GTGTTCATCTTTGGTTTTGTGGGCAACATGCTGGTCAT**tca**CTCATCCTGATAAACTGCAAAAGGCTGAAGAGCATGACTGACATCTA	+2 (Δ1+3)	x2
	GTGTTCATCTTTGGTTTTGTGGGCAACATG---------------CTGATAAACTGCAAAAGGCTGAAGAGCATGACTGACATCTACC	Δ15	x2
ZFNA	GGTTCTGGGTACTTTTATCTGTCCCCTCCACCCCACAGTGGGGCCACTAGGGACAGGATTGGTGACAGAAAAGCCCCATCCTTAGGCC	WT	
	GGTTCTGGGTACTTTTATCTGTCCCCTCCACCCCACAGTGGGGCCA**gcca**CTAGGGACAGGATTGGTGACAGAAAAGCCCCATCCTTA	+4	x17
	GGTTCTGGGTACTTTTATCTGTCCCCTCCACCCCACAGTGGG**cca**GCCACTAGGGACAGGATTGGTGACAGAAAAGCCCCATCCTTAG	+3	x6
	GGTTCTGGGTACTTTTATCTGTCCCCTCCACCCCACAGT----CCACTAGGGACAGGATTGGTGACAGAAAAGCCCCATCCTTAGGCC	Δ4	x4
	GGTTCTGGGTACTTTTATCTGTCCCCTCCACCCCACAGTGGG----------ACAGGATTGGTGACAGAAAAGCCCCATCCTTAGGCC	Δ10	x3
	GGTTCTGGGTACTTTTATCTGTCCCCTCCACCCCACAG----GCCACTAGGGACAGGATTGGTGACAGAAAAGCCCCATCCTTAGGCC	Δ4	x2
	GGTTCTGGGTACTTTTATCTGTCCCCTCCACCCCACAGTG---CCACTAGGGACAGGATTGGTGACAGAAAAGCCCCATCCTTAGGCC	Δ3	x2
	GGTTCTGGGTACTTTTATCTGTCCCCTCCACCCCACA**a**TGGGGCCA**gcca**CTAGGGACAGGATTGGTGACAGAAAAGCCCCATCCTTA	+4 (Δ1+5)	x2
	GGTTCTGGGTACTTTTATCTGTCCCCTCCACCCCACAGTGGGGCCAC**cac**TAGGGACAGGATTGGTGACAGAAAAGCCCCATCCTTAG	+3	x2
	GGTTCTGGGTACTTTTATCTGTCCCCTCCACCCCACAGTGG----ACTAGG-ACAGGATTGGTGACAGAAAAGCCCCATCCTTAGGCC	Δ5	x2
TA04	GGTTCTGGGTACTTTTATCTGTCCCCTCCACCCCACAGTGGGGCCACTAGGGACAGGATTGGTGACAGAAAAGCCCCATCCTTAGGCC	WT	
	GGTTCTGGGTACTTTTATCTGTCCCCTCCACCCCACAGTGGG----------ACAGGATTGGTGACAGAAAAGCCCCATCCTTAGGCC	Δ10	x6
	GGTTCTGGGTACTTTTATCTGTCCCCTCCACCCCACAGTGG---CACTAGGGACAGGATTGGTGACAGAAAAGCCCCATCCTTAGGCC	Δ3	x4
	GGTTCTGGGTACTTTTATCTGTCCCCTCCACCCCACAGTG----CACTAGGGACAGGATTGGTGACAGAAAAGCCCCATCCTTAGGCC	Δ4	x4
	GGTTCTGGGTACTTTTATCTGTCCCCTCCACCCCACAGTGGGG---------ACAGGATTGGTGACAGAAAAGCCCCATCCTTAGGCC	Δ9	x4
	GGTTCTGGGTACTTTTATCTGTCCCCTCCACCCCACAGT----CCACTAGGGACAGGATTGGTGACAGAAAAGCCCCATCCTTAGGCC	Δ4	x3
	GGTTCTGGGTACTTTTATCTGTCCCCTCCACC--ACAGT----CCACTAGGGACAGGATTGGTGACAGAAAAGCCCCATCCTTAGGCC	Δ6	x3
	GGTTCTGGGTACTTTTATCTGTCCCCTCCACCCCACAGT-----CACTAGGGACAGGATTGGTGACAGAAAAGCCCCATCCTTAGGCC	Δ5	x3
	GGTTCTGGGTACTTTTATCTGTCCCCTCCACCCCACAGTGG---CACTAGG-ACAGGATTGGTGACAGAAAAGCCCCATCCTTAGGCC	Δ4	x3
	GGTTCTGGGTACTTTTATCTGTCCCCTCCACCC-ACAGT-----CACTAGGGACAGGATTGGTGACAGAAAAGCCCCATCCTTAGGCC	Δ6	x2
	GGTTCTGGGTACTTTTATCTGTCCCCTCCACCCCACAGTGGGGC**ggc**CACTAGGGACAGGATTGGTGACAGAAAAGCCCCATCCTTAG	+3	x2
	GGTTCTGGGTACTTTTATCTGTCCCCTCCACCCCACAGTG-----ACTAGGGACAGGATTGGTGACAGAAAAGCCCCATCCTTAGGCC	Δ5	x2
	GGTTCTGGGTACTTTTATCTGTCCCCTCCACCCCAC-----------TAGGGACAGGATTGGTGACAGAAAAGCCCCATCCTTAGGCC	Δ11	x2
	GGTTCTGGGTACTTTTATCTGTCCCCTCCAC----------------TAGGGACAGGATTGGTGACAGAAAAGCCCCATCCTTAGGCC	Δ16	x2
	GGTTCTGGGTACTTTTATCTGTCCCCTCCACC--ACAGT-----CACTAGG-ACAGGATTGGTGACAGAAAAGCCCCATCCTTAGGCC	Δ8	x2
	GGTTCTGGGTACTTTTATCTGTCCCCTCCACCCCACAGTGGG---ACTAGG-ACAGGATTGGTGACAGAAAAGCCCCATCCTTAGGCC	Δ4	x2
	GGTTCTGGGTACTTTTATCTGTCCCCTCCACCCCACAGTGGG---ACTAG--ACAGGATTGGTGACAGAAAAGCCCCATCCTTAGGCC	Δ5	x2
	GGTTCTGGGTACTTTTATCTGTCCCCTCCACCCCACAGTGGGGC-ACTA---ACAGGATTGGTGACAGAAAAGCCCCATCCTTAGGCC	Δ4	x2
	GGTTCTGGGTACTTTTATCTGTCCCCTCCACCCCACAGTGG-----------ACAGGATTGGTGACAGAAAAGCCCCATCCTTAGGCC	Δ11	x2
	GGTTCTGGGTACTTTTATCTGTCCCCTCCACCCCACAGTGGGGCCAC-------AGGATTGGTGACAGAAAAGCCCCATCCTTAGGCC	Δ7	x2

[1] The nuclease target sites are underlined. Insertions are indicated as lower bold letters, deletions with --.

Supplementary Table S7. List of primers used in this study

Primer ID	Sequence (5'…3')	Target	Application
#985	aagatggattatcaagtgtcaagtcc	CCR5	T7E1-Del/Inv
#986	caaagtcccactgggcg	CCR5	T7E1-Del/Inv
#1093	tcagtgaagggagcagtgtg	IL2RG	T7E1
#1094	aacaacacgctaacccaacc	IL2RG	T7E1
#1185	CCCCTTACCTCTCTAGTCTGTGC	AAVS1	T7E1
#1186	CTCAGGTTCTGGGAGAGGGTAG	AAVS1	T7E1
#1474	ggcaaagactgggaagttgc	CCR2	T7E1-Del/Inv
#1475	GGGACAGAAGCAAACACAGC	CCR2	T7E1-Del/Inv
#1479	agccacatcgctcagacac	GAPDH	RT-PCR
#1480	gccaatacgaccaaatcc	GAPDH	RT-PCR
#1570	ccagaagagctgagacatcc	CCR5	RT-PCR
#1571	gctgcgatttgcttcacattg	CCR5	RT-PCR
#1574	gagcacttggtgcagtaccg	IL2RG	RT-PCR
#1575	cgaacacgaaacgtgtagcg	IL2RG	RT-PCR
#1714	CCAACGCCGACGGTATCAG	AAVS1	RT-PCR
#1716	GCGATATCTAGGTAGCCACAGG	AAVS1	RT-PCR
003-046414377F	GCACAGGGTGGAACAAGATGG	CCR5	SMRT
003-046414377R	ACCACCCCAAAGGTGACCGT	CCR5	SMRT
003-046399065F	TTGAACAAGGACGCATTTCCCCAG	CCR2	SMRT
003-046399065R	CAAAGACCCACTCATTTGCAGCAG	CCR2	SMRT
007-069824094F	GGGAAAATTGCAGCTGAAGGACC	AUTS2	SMRT
007-069824094R	CCTGGAAGCAGATCCCTCAAG	AUTS2	SMRT
016-062697711F	ACTGAATGGACCCCTCGTGG	CDH8	SMRT
016-062697711R	GGCTTTGCAGGGCCATTTCAAAC	CDH8	SMRT
002-049998047F	GGGCTTTGAGAGGTAATGAGGG	NRXN1	SMRT
002-049998047R	TACCTGGCTGTGCACAGTGG	NRXN1	SMRT
006-055393767F	GGCTACCCAAATGGATCTGTGAG	HMGCLL1	SMRT
006-055393767R	CCACTCTGTGCAAGTTGGCTTAC	HMGCLL1	SMRT
001-017587659F	AGACACCCATGTCAGGAGGGA	PADI3	SMRT
001-017587659R	CTGGGCCTCAATTTCCTCAACTG	PADI3	SMRT
006-005894754F	TTGTATCAGGCTGGCCAGGG	NRN1	SMRT
006-005894754R	CAAGGACAGATGTTCCTTCCACTC	NRN1	SMRT
00X-012827277F	CTCATGTACGTCTTAGTGGGAGG	PRPS2	SMRT
00X-012827277R	GGTGGTGATAGAGACTGGAAGTAC	PRPS2	SMRT
001-090419868F	AAAAAACCCAGGCATGGGGCTAG	LRRC8D	SMRT
001-090419868R	AGTGAGGGCTGATGGCTGAG	LRRC8D	SMRT
015-058836113F	GCCGGCCATGAGGACATTTTTAC	LIPC	SMRT
015-058836113R	TGGAGAGGGTTCTGGGCCTT	LIPC	SMRT
021-044089791F	GGCCTCAGTTTCAACATCAGTGAC	PDE9A	SMRT
021-044089791R	AAGGTGATGCTGGCATGCCC	PDE9A	SMRT
003-039373855F	AGTGACCGACTACTACTACCCTG	CCR8	SMRT
003-039373855R	AACACCCACTGGTCCAGCAGA	CCR8	SMRT
011-082667886F	GCCACCCCACTTTCTGCTAATC	C11orf82	SMRT
011-082667886R	GCACTGAACTTGAGAGCAAGTACC	C11orf82	SMRT
007-036783485F	CAAGGAAGGATAGGCTGGAGATCA	AOAH	SMRT
007-036783485R	CCGAGAGGTTATGGGGTTCC	AOAH	SMRT
012-019068737F	CCTGCCTTTTCAAGTGACTGACTG	CAPZA3	SMRT
012-019068737R	GTGAGTGGTTTTGAGGACTGCTG	CAPZA3	SMRT
010-007305407F	TTAGCCAGGCTGGTCTCGAAC	SFMBT2	SMRT
010-007305407R	TTTGGCCTTCAGGTGGGACACT	SFMBT2	SMRT
002-014773961F	ACGTAAGCTAGCCGGCAGGT	FAM84A	SMRT

002-014773961R	ACGCCAAACTTGTCGGGCTG	FAM84A	SMRT
00X-009227571F	CCAAGATCTGTGAGCTTAGCATGC	TBL1X	SMRT
00X-009227571R	TTGTGGGCAGAGTAGTATCTGACC	TBL1X	SMRT
006-107261416F	TGCCCTCTCTCACCACTCCT	LOC100422737	SMRT
006-107261416R	CTCAGCTTGACTGTTGGTGGTG	LOC100422737	SMRT
012-089421427F	GCATTCACTCGCCTGTTCCTC	LOC728084	SMRT
012-089421427R	CTAGGAGAAGGTTCAAAGGCCTG	LOC728084	SMRT
010-127381108F	GACACCACCATCCTTGGGGAA	LOC283038	SMRT
010-127381108R	GGACTCACAAGGACAGGCTC	LOC283038	SMRT
022-041625653F	TGGCGAGAGTAAGAGCCACC	CHADL	SMRT
022-041625653R	ACCACCCTGCCAAGCACTAG	CHADL	SMRT
004-008528028F	GTCAGTCGATGAGCTGGGATCT	TRMT44	SMRT
004-008528028R	TAAGGAGGCTGGAGGGAGCA	TRMT44	SMRT
006-114639685F	CCCACTAAGGCTCTTCACAGCA	HS3ST5	SMRT
006-114639685R	GGTCCAAGCATAGTGCTGAGTG	HS3ST5	SMRT
003-173622983F	GGTCTAGTCCCACAGATTCCAAC	NLGN1	SMRT
003-173622983R	GCCACATGAGAGGTCTCTCTTC	NLGN1	SMRT
007-076237892F	ACATGTCTGTACCCGAGTCAACAC	LOC100133091	SMRT
007-076237892R	ATCCCTCAGTAGCTGGCTCC	LOC100133091	SMRT
008-084679015F	GACAAGAATGCCCTCATTCACCAC	RALYL	SMRT
008-084679015R	CCTATCCATGAGCATGACCAAGAG	RALYL	SMRT
011-066963668F	ATCCATGGTGCACGAGCAGG	KDM2A	SMRT
011-066963668R	CATTCAGCCAAGCACTGTGGCTC	KDM2A	SMRT
011-013485053F	CCTTTGAATCCCACGTTTTCCCC	BTBD10	SMRT
011-013485053R	TTTCAGCTGGCACAGGGGTTC	BTBD10	SMRT
008-073736694F	AGGCATCTCGAGGTGTGCCA	KCNB2	SMRT
008-073736694R	GACACTCAGCTTTGTCTCTGCAAC	KCNB2	SMRT
007-070919207F	TCCCCCAGACTCCACCAACA	WBSCR17	SMRT
007-070919207R	GGGAAGGCTGCAACCATTTATGAG	WBSCR17	SMRT
012-075963284F	CCACAGAGAGAGACACCATCTCA	KRR1	SMRT
012-075963284R	AGAGATGGGGGTTTCACCATGTTG	KRR1	SMRT
004-104555586F	CCCAGTAGGTGGTGCAGCTAT	TACR3	SMRT
004-104555586R	GCTATGGATACTGAGAGACTGG	TACR3	SMRT
019-055627003F	CCAGGAAATGGGGGTGTGTC	AAVS1	SMRT
019-055627003R	GTCCACTTCAGGACAGCATGTTTG	AAVS1	SMRT
008-141506929F	ATAGCATTGCCACCAGCAGTGTGT	CHRAC1	SMRT
008-141506929R	CAGCTATTCGGGAGGCTGAG	CHRAC1	SMRT
010-047635249F	CTGAAGACACCATGTCTCATGCC	ANXA8L2	SMRT
010-047635249R	CTGGATCAGGAAGAGCAGGAG	ANXA8L2	SMRT
004-003303328F	ATGGAGAGTGAGGCCGGGGT	RGS12	SMRT
004-003303328R	AAACCTGGCTCATTCCAACGTCC	RGS12	SMRT
010-117758576F	GAAGGTGGTGCTTAACCTTCCAG	ATRNL1	SMRT
010-117758576R	GGCACTGTATGGCAAAGGACC	ATRNL1	SMRT
014-101033005F	ACCTGGACCTTGGCCACGACA	BEGAIN	SMRT
014-101033005R	CTTAGTGCCGTGGGTGAGTG	BEGAIN	SMRT
013-040779804F	CCTGTGGTGAGATAGCACCTCTAA	LINC00548	SMRT
013-040779804R	TATCTGGGGTGGCCCACAAAAG	LINC00548	SMRT
010-044697899F	CCTCCACAACCATGAGTGACATC	LOC100130539	SMRT
010-044697899R	CAACCCCTCTCCAGACCTCA	LOC100130539	SMRT
010-126241867F	AACCCAAGTGGGGTTCTCATGTG	LHPP	SMRT
010-126241867R	AGAATGGCGTGAACCCGGGA	LHPP	SMRT
002-234242554F	CAGCTGCTTTTACCTATCCAGGG	SAG	SMRT
002-234242554R	ATCAACCCCATACCAAGCAGGTG	SAG	SMRT
003-071561247F	TACGTGAGAAAACCACGGCTCAG	FOXP1	SMRT
003-071561247R	ACCTTGCTTACAGGGTGTGTCTGC	FOXP1	SMRT

011-002046928F	AACCTCAGGCACCCCTCAAC	H19	SMRT
011-002046928R	GTCCTCCCTTTTAGCATCTACCAG	H19	SMRT
005-149981775F	TTACTGGGCGCCAGGCTCT	SYNPO	SMRT
005-149981775R	GCTGGCTTGATATGCTATTTAGACACCTTTC	SYNPO	SMRT
001-003439947F	AGCCCTGTGCCGGTTCCTA	MEGF6	SMRT
001-003439947R	GCTGCGTGAGGACACCCTT	MEGF6	SMRT
016-066569433F	GGATTCTTTATTGAAAGACTGAGAGGTTTTGCC	TK2	SMRT
016-066569433R	TGCCTAGCACTTAGTAGGGGCTT	TK2	SMRT
018-022999683F	TGCTTCTGTGTGCAGGAAGAGG	ZNF521	SMRT
018-022999683R	CTTTGTCGGGTACAGCGGACAAT	ZNF521	SMRT
015-074546864F	GGAGTGACCTCCCAATGGCT	CCDC33	SMRT
015-074546864R	CTGGGTGACAAGAGTGAAACTCC	CCDC33	SMRT
012-101079037F	GCAAGAGATGGGCTCCCACA	GAS2L3	SMRT
012-101079037R	CCCTCATGGAGAACCTCTGC	GAS2L3	SMRT
011-011689575F	CCCTTCTGAGCATTTGACACAGGT	MIR4299	SMRT
011-011689575R	ACCTACAGGAGTGGGTACAGC	MIR4299	SMRT
005-001180652F	CTTGGGGAACCCATTGCCGA	SLC6A19	SMRT
005-001180652R	ACCAGGCACCTGGATGCATG	SLC6A19	SMRT
010-119572269F	CAGCCTTGGCCAACAACACAC	RAB11FIP2	SMRT
010-119572269R	TTGCTTGAGCCCAGTAGGTGGA	RAB11FIP2	SMRT
010-015227350F	AATCCCTTCAGTAGACCCACACAG	NMT2	SMRT
010-015227350R	GGTGGGGCAGGCTGTTTTGT	NMT2	SMRT
008-027446593F	TCAGAGCAGAAAACAAGCTACCCTCATG	CLU	SMRT
008-027446593R	CAGGGCTCTTCTTTCTCCTGG	CLU	SMRT
006-041727666F	ACTCTTCCCAACATCCCAACATCC	FRS3	SMRT
006-041727666R	CCAGACTTGGCAGGAAACAGG	FRS3	SMRT
019-055627003F	CCAGGAAATGGGGGTGTGTC	AAVS1	SMRT
019-055627003R	GTCCACTTCAGGACAGCATGTTTG	AAVS1	SMRT
022-051139409F	CAGAGCCTCTGCTCTGGGAT	SHANK3	SMRT
022-051139409R	GGAGACTGCTCTCTGCTGGGT	SHANK3	SMRT
017-035084318F	TTTACGAGCCGGGAGCCTCT	MRM1	SMRT
017-035084318R	GCAGGGGAGAGGGGAAAAAAG	MRM1	SMRT
016-065705732F	TCACTTCTGCTCCTCTGCCTG	LOC283867	SMRT
016-065705732R	CAGGCTTCTTACATGGCAGCTC	LOC283867	SMRT
007-100769506F	CAAGCTGCCCCAAGTCCTAG	SERPINE1	SMRT
007-100769506R	GGACTCTTGGTCTTTCCCTCATC	SERPINE1	SMRT
002-155321765F	TACTCTCCTCTACCCCCCAC	LOC100144595	SMRT
002-155321765R	GGTTCTAGTAAGGGATTTAGGGTGCATG	LOC100144595	SMRT
019-041319015F	CCCAAATGTTCTGTGCCCATGC	RAB4B-EGLN2	SMRT
019-041319015R	TTTGGCCAAGCAGGGACAGG	RAB4B-EGLN2	SMRT
00X-018438371F	CTCAGGTCATGACCATCAGTGATC	CDKL5	SMRT
00X-018438371R	GCTTGTGGCTCATGGCACCT	CDKL5	SMRT
007-043424775F	ATCACTTGGCAGTCTCCCCTC	HECW1	SMRT
007-043424775R	GGTGAGTCCAGTGTATACTCAAGG	HECW1	SMRT
010-101836421F	CTGAACAAGAACACAGCAGGTGAG	CPN1	SMRT
010-101836421R	TGGCCCAAGAGAAGACTGAGG	CPN1	SMRT
016-027592693F	CATCTTTTAACAGTGCATGAAAGGGATAGAGC	KIAA0556	SMRT
016-027592693R	TTTTACCAAATACCACCGGTTCCCCC	KIAA0556	SMRT
001-015490179F	GTCCAGAGATGGTCCTAGCC	TMEM51	SMRT
001-015490179R	GGAGGCTGAGGCAGGAGAAT	TMEM51	SMRT
017-038076372F	TCGTTGTTGATGTTCTCCAGCACC	ORMDL3	SMRT
017-038076372R	ACAAAGTCCCAGGTAGAAGGGAG	ORMDL3	SMRT
004-012840270F	CATTTAACAAGAGAGACAACTGAGGCACAGA	HSP90AB2P	SMRT
004-012840270R	AGGTGGGCTGAGAGCTCACA	HSP90AB2P	SMRT
010-120434186F	GAGTCACTGCCTCACTGGATTG	CACUL1	SMRT

010-120434186R	TTCTTGGGTATGTCCAGATTCACCCAAAAC	CACUL1	SMRT
001-118483616F	GAATTGCAGATTTTTAGGGAATCATCACTTTAGGG	WDR3	SMRT
001-118483616R	CTAGTCCTTGTTATTTTCTCCTAAGTATGGTACTC	WDR3	SMRT
006-033425103F	GAACAACTTACCTTGGTCAGAGGG	ZBTB9	SMRT
006-033425103R	CCATTCTCACATTCTCTCCCACAC	ZBTB9	SMRT
009-033366297F	ATTTTTGGGGAATATCACCCACTCCCCAG	NFX1	SMRT
009-033366297R	CTAATCTTACCACACAAGAATTGCAGGGATC	NFX1	SMRT
002-026928507F	CACAGAGGGTTGTGTTGGCC	KCNK3	SMRT
002-026928507R	AGCACACAAGGGCCGTCTGA	KCNK3	SMRT
001-221693221F	CACCTGTCACAATTCTGGAGCAAC	DUSP10	SMRT
001-221693221R	CTAGGTGCTATCTCTCCCCTG	DUSP10	SMRT
006-105034730F	TTTGCCTGAAGCCTTTAATTTTCCACAAGCC	HACE1	SMRT
006-105034730R	TAATGACCTGCTGTGTACAACTGCTTAAAGGA	HACE1	SMRT
016-017287252F	AGTTGGTGGTGGGGCAGAATC	XYLT1	SMRT
016-017287252R	AGTGCCCAAGTCAGGCACTTC	XYLT1	SMRT
009-130229769F	TGTCCGGCTCTCTCTCTGCT	LRSAM1	SMRT
009-130229769R	GAGGCGTCAAGGCTCAGCAT	LRSAM1	SMRT
